# Imaging of macrophage mitochondria dynamics *in vivo* reveals cellular activation phenotype for diagnosis

**DOI:** 10.7150/thno.40495

**Published:** 2020-02-03

**Authors:** Yue Li, Yuan He, Kai Miao, Ying Zheng, Chuxia Deng, Tzu-Ming Liu

**Affiliations:** 1 Institute of Translational Medicine, Faculty of Health Sciences, University of Macau, Taipa, Macao SAR, China; 2 Institute of Chinese Medical Sciences, University of Macau, Taipa, Macao SAR, China

**Keywords:** macrophage, mitochondria, liposome, *in vivo* imaging, multiphoton microscopy

## Abstract

Highly plastic macrophages are pivotal players in the body's homeostasis and pathogenesis. Grasping the molecular or cellular factors that drive and support the macrophage activation will help to develop diagnostics and manipulate their functions in these contexts. However, the lack of *in vivo* characterization methods to reveal the dynamic activation of macrophages impedes these studies in various disease contexts.

**Methods**: Here,* in vitro* bone marrow-derived macrophages (BMDMs) and *in vivo* Matrigel plug were used to evaluate how mitochondria dynamics supports cellular activation and functions. We conducted macrophage repolarization *in vitro* to track mitochondria dynamics during the shift of activation status. For *in vivo* diagnosis, a novel MitoTracker-loaded liposome was first developed to label macrophage mitochondria in mice before/after inflammatory stimulation.

**Results**: Based on the typical activation of *in vitro* BMDMs, we found glycolysis based macrophages have punctate and discrete mitochondria, while OXPHOS active macrophages have elongated and interconnected mitochondria. M1, M2a, M2b, and M2c activated BMDMs showed clustered and differentiable features in mitochondrial morphology. These features also hold for Matrigel plug-recruited macrophages in mice. Furthermore, with the interventions on M2a macrophages *in vitro*, we demonstrated that mitochondria morphology could be a metabolic index to evaluate macrophage activation status under drug manipulation. Using the MitoTracker-loaded liposomes, we further achieved subcellular imaging of macrophage mitochondria* in vivo*. Their organization dynamics revealed the dynamic change from anti-inflammatory macrophages to inflammatory ones* in vivo* under the lipopolysaccharide (LPS) challenge.

**Conclusion**: These results reveal that subcellular imaging of mitochondria organization can characterize the activation status of macrophage *in vitro* and *in vivo* at a single-cell level, which is critical for the studies of noninvasive diagnosis and therapeutic drug monitoring.

## Introduction

Macrophages, a type of immune cell, have remarkable plasticity and can modify their physiology to carry out tissue-dependent functions when exposed to special environmental cues. Activated macrophages are commonly classified as pro-inflammatory (M1) and alternatively activated (M2) macrophages, and M2 macrophages can be further subdivided into M2a, M2b, M2c, and M2d sub-types based on the stimulation scenarios 1. Accumulating evidence indicates that abnormally activated macrophages are closely related to a plethora of diseases, including cancer, diabetes, obesity, and atherosclerosis 2. Therefore, a better understanding of the molecular or cellular factors that drive and support the macrophage activation will help to grasp and manipulate their functions in these contexts.

Recent immunometabolism studies conclude that metabolic shifts triggered by activation scenarios support the functions of activated macrophages 3. For instance, M1 macrophages enhance glycolysis, fatty acid *de novo* synthesis, and pentose phosphate pathway (PPP) to support pro-inflammatory and microbial killing function. M2a macrophages augment oxidative phosphorylation (OXPHOS) and fatty acid oxidation (FAO) to promote tissue remodeling and repairing. These characteristic metabolic signatures not only provide an opportunity to identify activated macrophages from the metabolomic assay 4 and metabolic imaging 5-7, but also provide a therapeutic target to edit macrophage polarization by manipulating their metabolism 8. For example, Chen and colleagues used chloroquine to repolarize M2-like tumor-associated macrophages (TAMs) toward the pro-inflammatory M1 phenotype by driving their metabolic shift from OXPHOS to glycolysis. Chloroquine-reset macrophages inhibited tumor development by ameliorating immunosuppression 9. Conversely, inactivation of pro-inflammatory macrophages may stop the cytokine storms, tissue damage, or chronic inflammation 10. However, the re-activation of M1 macrophages toward healing-promoted M2 states in an inflammatory environment remains a challenge. Recently, Bossche and colleagues showed that inflammatory M1 macrophages disable their mitochondrial function to impede the repolarization to an anti-inflammatory M2 phenotype 11. These findings suggest that activated macrophages might modify mitochondria biology to maintain their function and determine the cell fate.

On the other hand, recent studies also revealed that mitochondrial structures are highly dynamic and correlated with cell lineages 12. Their organizations could determine the metabolic function of cells and are mainly regulated by the concentration ratio of fusion proteins over fission ones. Dynamin-related protein 1 (Drp1) causes mitochondrial fission when activated by kinases, producing fragmented and discrete mitochondria 13. Mitochondrial fragmentation can impair OXPHOS 14, augment reactive oxygen species (ROS) generation 15, and facilitate mitophagy 16. Mitochondrial fusion includes mitofusin (Mfn1 and Mfn2) mediated outer membrane fusion and optic atrophy 1 (Opa-1) mediated inner membrane fusion, driving mitochondria into elongated and interconnected networks. Mitochondrial fusion can maximize OXPHOS activity by remodeling cristae shape 17, 18, and prolong cell longevity 19. Recently, Buck and colleagues discovered that enforcing mitochondrial fusion in effector T cells improves antitumor function by elevating their OXPHOS capacity and longevity 20. This study indicated that mitochondrial morphology is strongly correlated to cell-type due to its metabolic shift. Such a morphological phenotype of cellular metabolism could serve as an index for cell-type identification.

Although past research has revealed that mitochondrial organization correlates macrophage function 21, 22, there are limited studies to analyze the mitochondrial morphology in different subtypes of activated macrophages, not to mention to use the extracted features to characterize their activation status. In this study, we use bone marrow-derived macrophages (BMDMs) to assess the mitochondrial morpho-dynamics during macrophage maturation and activation. Through the morphological analysis of the mitochondrial organization, we extract the characteristic parameters that can differentiate and identify the activation status of macrophages. With these parameters and local delivery of liposome-encapsulated MitoTracker, we successfully realized the targeted labeling of macrophage mitochondria and the *in vivo* characterization of macrophage activation in the context of inflammation challenges. Thus developed approach can help to identify macrophage metabolic phenotype and activation status at the single-cell level in the dynamic immune microenvironment (such as tumors, wound, and diabetic adipose tissues), which is pivotal for disease diagnosis and macrophage-targeted drug evaluation.

## Materials and Methods

### Mice

C57BL/6 mice were obtained from the Animal Facility of the Faculty of Health Sciences at the University of Macau. B6 (Cg)-Tyr^c-2J^/J transgenic mice and B6.129P2-Lyz2^tm1(cre)Ifo^/J transgenic mice were purchased from The Jackson Laboratory. STOCK Gt(ROSA)26Sor^tm4(ACTB-tdTomato,-EGFP)Luo^/J transgenic mice were kind gifts from Dr. Kai MIAO (University of Macau). The Animal Research Ethics Committee of the University of Macau approved all experiments.

### Generation of macrophage-reporter mice

B6.129P2-Lyz2^tm1(cre)Ifo^/J mice express Cre from the myeloid-specific LysM promoter. STOCK Gt(ROSA)26Sor^tm4(ACTB-tdTomato,-EGFP)Luo^/J mice are dual-reporter (membrane-targeted Tomato [mTOM]/ membrane-targeted enhanced green fluorescent protein [mEGFP]-floxed reporter) mouse strain. This strain expresses reporter gene mTOM (red) in the absence of Cre, whereas expresses reporter gene mEGFP (green) upon Cre activation. B6.129P2-Lyz2^tm1(cre)Ifo^/J mice were crossed with STOCK Gt(ROSA)26Sor^tm4(ACTB-tdTomato,-EGFP)Luo^/J mice to get LysM-Cre\mTmG mice in which LysM-Cre positive myeloid cells would express EGFP.

### Mononuclear cell isolation

Whole blood of C57BL/6 mice was collected from retro-orbital venous plexus using heparinized (Sigma) microtubes. The heparinized blood was diluted with the four-times volume of PBS containing 0.5% bovine serum albumin (BSA), then layered on 4 mL Histopaque (1.077g/mL, Sigma) gradient layer. Mononuclear cells were collected from the 1.077-PBS interface. After the elimination of red blood cells (RBCs) via hypotonic lysis, mononuclear cells were resuspended in the completed RPMI-1640 medium for mitochondria staining and the next examination. PE anti-mouse CD115 (CSF-1R) antibody (AFS98, 1:80, Biolegend) was used to gate monocytes from mononuclear cells.

### Macrophage preparation and drug treatments

Bone marrow-derived macrophages (BMDMs) were differentiated from mouse bone marrow cells 23. Briefly, bone marrow cells were isolated from the femur and tibia of C57BL/6 mice (or LysM-Cre\ mTmG mice) and cultured in the completed RPMI- 1640 medium containing 20 ng/mL recombinant murine M-CSF (rmM-CSF, 315-02, PeproTech) or 15% L929 cells (ATCC) conditioned medium. On day 6, cells were harvested from Petri dishes by 20 mM EDTA treatment for further examination.

For macrophage activation, BMDMs were stimulated for 24 hr with certain cytokines after removal of rmM-CSF. 20 ng/mL IFN-γ (315-05, PeproTech) plus 10 ng/ml LPS (O111:B4, Sigma-Aldrich) was used to induce M1 macrophages, 20 ng/mL IL-4 (214-14, Peprotech) to induce M2a, 10 µg/mL immune complex (150 μg/ml monoclonal anti-chicken egg albumin antibody [C6534] preincubated with 15 μg/ml ovalbumin [A5503] at 37°C for 30 min; Sigma Aldrich) plus 50 ng/ml LPS (O111:B4, Sigma-Aldrich) to induce M2b, and 50 ng/mL IL-10 (210-10, Peprotech) to induce M2c, respectively.

For repolarization of M2a macrophages, BMDMs were primed with IL-4 for 24 hr to induce M2a macrophages; then cells were washed and treated with IFN-γ plus LPS for an additional 24 hr. To investigate the blocking of macrophages toward M1 polarization, the BMDMs were stimulated for 24 hr with IFN-γ plus LPS either in the presence or absence of 10 µM Mdivi-1 (Sigma-Aldrich) plus 20 µM fusion promoter M1 (Sigma-Aldrich), 100 µM 1400W (Beyotime), or 5 mM NAC (Sigma-Aldrich).

### Flow Cytometry

Nonspecific binding was blocked by Fc-block (Mouse TruStain FcX™ PLUS, S17011E, 1:2000, Biolegend) before surface staining. Fluorescent-dye conjugated monoclonal antibodies were used to identify macrophage maturation and to differentiate their activation status, including Alexa Fluor 647 anti-mouse F4/80 antibody (BM8, 1:200, Biolegend), PE anti-mouse/human CD11b antibody (M1/70, 1:80, Biolegend), PE anti-mouse CD86 antibody (GL-1, 1:20, Biolegend), PE anti-mouse CD206 antibody (C068C2, 1:40, Biolegend), and PE anti-mouse CD163 antibody (TNKUPJ, 1:80, eBioscience).

Mitochondria staining was performed according to the manufacturer's instructions (M7514, 100 nM, Invitrogen). Briefly, cells were incubated in the completed medium containing 100 nM MitoTracker Green FM for 30 min; then cells were washed twice with the completed medium. Cell viability was assessed by 7-AAD staining (1:100, Biolegend).

JC-1 staining was performed according to the manufacturer's instructions (ab113850, Abcam). Briefly, cells were incubated in completed medium containing 5 μM JC-1 for 20 min; then cells were washed twice with the completed medium. 100 μM fluoro-carbonyl cyanide phenylhydrazone (FCCP)-treated cells were used as a positive control.

For the staining of intracellular arginase, cells were fixed and permeabilized with 100% methanol (chilled at -20°C) at room temperature for 5 min and were stained with anti-mouse arginase-1 antibody (24HCLC, 1:100, Invitrogen), followed by incubation with Alexa Fluor 488 Donkey anti-Rabbit Secondary Antibody (A-21206, 1:200, Invitrogen).

For the staining on macrophages of mice ear, a single-cell suspension was prepared as described before 24. Briefly, mice ear was chopped with scissors into small pieces and post immersed in the digestion cocktail (2.5 mg/ml Collagenase Type 2 [Worthington-Biochem], 2.5 mg/ml Collagenase Type IV [Gibco] and 0.5 mg/ml DNAse I [Roche]) at 37 °C for 90 min. Following incubation, digested skin samples were squeezed and filtered to get the suspension of cells. Data were acquired with a BD Accuri™ C6 Cytometer (BD Biosciences, USA) and analyzed using BD Accuri C6 Software (BD Biosciences) or FlowJo software (Tree Star).

### Transmission electron microscopy (TEM)

BMDMs were stimulated as described above. Cells were first fixed with 2.5% glutaraldehyde at 4 °C overnight. Following fixation, cells were washed three times with PBS and postfixed in 1% osmic acid at 4 °C for 1.5 hours. Then cells were dehydrated in a graded ethanol series and embedded in acrylic resin. Cut sections were stained with uranyl acetate and lead citrate, and then imaged using a Hitachi H-7500 transmission electron microscope (Hitachi, Japan) equipped with a digital camera.

### Metabolism Assays

XF-96 Extracellular Flux Analyzer (Seahorse Bioscience, USA) was used to analyze the metabolic profiles of activated macrophages 4. 25,000 BMDMs were seeded in a 96 well culture plate and activated as indicated. Oxygen consumption rates (OCR) and extracellular acidification rates (ECAR) were measured after the sequential addition of 1 µM oligomycin; 1.5 µM fluoro-carbonyl cyanide phenylhydrazone (FCCP); and 100 nM rotenone plus 1 µM antimycin A. Bradford assay (500-0205, Bio-rad) was conducted to normalize cell number after metabolic analysis 4. Basal respiration can be calculated as the difference between OCR before drug injection and lower OCR after rotenone plus antimycin A injection. ATP production can be calculated as the difference between basal OCR and lower OCR after oligomycin injection. Spare respiratory capacity can be established by the difference between basal OCR and the highest OCR after FCCP injection. Basal glycolysis can be calculated from the measurement of ECAR before drug injection. Glycolytic capability can be calculated from the measurement of ECAR after oligomycin injection. The glycolytic reserve can be calculated as the difference between higher ECAR after oligomycin injection and highest ECAR after FCCP injection.

### Glucose uptake assay

For glucose uptake capacity, BMDMs were starved in the glucose-free medium for 1 hr, then incubated in glucose-free medium containing 100 µM 2-NBDG (Invitrogen) for 30 min in 37 °C incubator. Cell viability was assessed by 7-AAD staining (1:100, Biolegend). Data were acquired with a BD Accuri™ C6 Cytometer (BD Biosciences, USA) and analyzed using BD Accuri C6 Software (BD Biosciences) or FlowJo software (Tree Star).

### Macrophage function assay

BMDMs seeded in a 96 well culture plate were stimulated as described above. Intracellular reactive oxygen species (ROS) production and nitric oxide (NO) production were measured using DCFH-DA and DAF-FM DA respectively, according to the manufacturer's instructions (Beyotime). After incubation, fluorescence was detected by Microplate Reader (Molecular Devices, USA) immediately.

### Confocal microscopy imaging and mitochondrial morphology analysis

Mitochondria staining was performed according to the manufacturer's instructions (M7512, Invitrogen). Briefly, BMDMs were incubated in the completed medium containing 100 nM MitoTracker Red CMXROS for 30 min and then 2 μg/mL Hoechst 33342 (Sigma Aldrich) for an additional 2 min. After incubation, cells were washed twice and replaced with the completed medium for live-cell imaging. For live-cell imaging, BMDMs on confocal dishes were placed in a micro-incubator system, which maintained 37 °C and 5% CO_2_ within a humidified environment. Images were acquired using a Nikon A1MP+ fluorescence confocal microscope (Nikon, Japan) with a water-immersed 40× and 1.15 NA objective. We acquired MitoTracker images at 561 nm excitation, and Hoechst images at 405 nm excitation. Images were subsequently imported into ImageJ software for analysis.

Analysis of mitochondrial morphology (Figure S1) was performed using the macro MiNA in Fiji software (ImageJ) 25. Briefly, the macro MiNA creates a skeleton of the mitochondrial network by several preprocessing steps (Figure S1A), then calculates several parameters reflecting mitochondrial morphology (Figure S1B), such as mean length of branches, mean network size (the mean number of branches per network), and mitochondrial footprints (mitochondrial coverage area). Three dimensional (3D) or two dimensional (2D) scatter plots were generated via Origin software (version 8.6).

### Structured illumination microscopy (SIM)

For the super-resolution imaging of mitochondria, BMDMs were incubated in the completed medium containing 100 nM MitoTracker Red CMXROS for 30 min. After incubation, cells were washed twice and replaced with the completed medium for live-cell imaging. Super-resolution images were acquired using a Nikon N-SIM system (Nikon, Japan) with an oil-immersed 100× and 1.49 NA objective. Images for live-cell imaging (live N-SIM) were taken at a single Z-plane. Images were captured using Nikon NIS-Elements and reconstructed using slice reconstruction in NIS-elements. Cells used for live-cell imaging were maintained in a 37 °C, 5% CO_2,_ and humidified environment.

For super-resolution imaging of single liposome, MitoTracker-loaded liposome and Matrigel were mixed and smeared on the confocal dish to immobilize the liposome. Liposomes were imaged with the same imaging conditions used in live-cell experiments.

### *In vivo* Matrigel plug assay

Matrigel plug implanted in C57BL/6 mice was used for macrophage *in vivo* activation. To induce activated macrophages in mice, 0.25 mL Matrigel (BD Biosciences) containing rmM-CSF (100 ng/mL)+PBS was used for naive macrophage differentiation, Matrigel containing rmM-CSF+LPS (10ng/mL) + IFN-γ (20ng/mL) for M1, Matrigel containing rmM-CSF+IL-4 (20 ng/mL) for M2a, Matrigel containing rmM-CSF+immune-complexes (10 μg/mL)+LPS (50 ng/mL) for M2b, Matrigel containing rmM-CSF+IL-10 (50ng/mL) for M2c, and Matrigel containing PBS only for control group. All procedures were completed on ice. C57BL/6 mice were anesthetized with isoflurane, then Matrigel mixture was subcutaneously injected into the inguinal areas of mice.

Five days after plug implantation, mice were euthanized by CO_2_ asphyxiation for Matrigel plug excision. For *ex vivo* live-cell imaging, Matrigel plugs were labeled by MitoTracker Red CMXRos and Hoechst 33342. Then they were placed in the micro-incubator system on the confocal microscope for further observation. MitoTracker images were collected at 561 nm excitation, and Hoechst images were collected at 405 nm excitation. For immunofluorescence staining, Matrigel plugs were OCT-embedded and sectioned for further examination.

### Mitotracker-loaded liposome preparation

Liposomes were prepared by the ethanol injection method as described before 26. Briefly, 10 mg phospholipids and 5 mg cholesterol were dissolved in 500 µL ethanol, then MitoTracker Red CMXRos stocking solution was added to the above ethanolic solution. The resulting organic phase was dropped in 4 mL PBS under 100 rpm/min stirring. Unloaded MitoTracker Red CMXRos and ethanol solvent were removed by ultracentrifugation of liposome suspension at 14,000 rpm for 1 hour. The obtained precipitates were dispersed in PBS and stored at 4°C. After dilution with water, the particle size and zeta potential of MitoTracker-loaded liposomes were measured by a Zetasizer Nano-ZS (Malvern Instruments, UK).

For *in vitro* testing, cells were incubated in a completed medium containing MitoTracker Red CMXRos-loaded liposome for 60 min. Then, cells were washed twice and replaced with the completed medium for live-cell imaging. To evaluate the MitoTracker release from liposome during the cell endocytosis process, we constructed the fluorescent green/red dyes double-loaded liposome by labeling the membrane of Mitotracker-loaded liposomes with the Green Fluorescent Cell Linker Kit, according to the manufacturer's instructions (Sigma). Briefly, precipitates of Mitotracker-loaded liposome was dispersed in Diluent C from the Kit and subsequently mixed with equal volumes of Diluent C containing PKH67 dye for 3 min at room temperature. Then, stop the staining by adding PBS containing 1% FBS and remove unloaded PKH67 by ultracentrifugation at 14,000 rpm for 1 hour. After re-suspension, cells were incubated in a completed medium containing MitoTracker and PKH67 double-loaded liposome for 60 min. For the control group, MitoTracker-loaded liposome and free PKH67 were sequentially stained for 60 min and for 3 min. Then, cells were washed twice and replaced with the completed medium for live-cell imaging.

### *In vivo* skin inflammation experiment and two-photon excited microscopy imaging

The same experimental protocol was used and performed on c2J mice and LysM-Cre\mTmG mice. To observe the mitochondrial structure of resident macrophages *in vivo*, mice were anesthetized by intraperitoneal injection of Avertin (25 µL of injection volume/g of body weight), then a single subcutaneous injection of MitoTracker-loaded liposomes (in 30 µL PBS) was administrated into the dorsal side of ear pinna with the hair removed. One day after liposome injection, mice were anesthetized by Avertin for mitochondria intravital imaging. To track the dynamics of mitochondria morphology after inflammatory stimulation, the LPS (30 µg in 30 µL PBS) was subcutaneously injected into the dorsal side of ear pinna after mice anesthetization. On the 3^rd^-day post LPS injection, MitoTracker-loaded liposomes (in 30 µL PBS) were subcutaneously injected into the ear of mice to label mitochondria *in vivo*. On the 4^th^-day post LPS injection, mice were anesthetized by Avertin for intravital imaging. Briefly, anesthetized mice ear pinnae were mounted on slides, immersed by glycerol and nipped by a cover glass.

Images were acquired using a Nikon A1MP+ microscope (Nikon, Japan) with a water-immersed 40× and 1.15 NA objective. Two-photon laser excitation was provided by an 800-1300 nm tunable femtosecond laser (Insight X3, Spectra-Physics). For c2J mice, mitochondrial images were acquired at 1120 nm excitation. The fluorescence of MitoTracker Red CMXRos and second harmonic generation (SHG) images in the ear of c2J mice were collected in the wavelength range of 604-679 nm and 506-593 nm, respectively. For LysM-Cre\mTmG mice, EGFP cell images were acquired at 970 nm excitation, and the fluorescence of EGFP and SHG was collected in the wavelength range of 506-593 nm and 415-485 nm, respectively. Mitochondrial images of LysM-Cre\ mTmG mice were acquired at 1180 nm excitation, and the fluorescence of MitoTracker Red CMXRos was collected in the wavelength range of 604-679 nm. The corresponding spectra were acquired with an Andor iDus 401 series Spectroscopy CCD (Andor, EU) coupled on the back-side port of the Nikon A1MP+ microscope and analyzed using Graphpad Prism software (version 6.0). For immunofluorescence staining, mice's ear was OCT-embedded and sectioned for further examination. For the flow cytometer, mice's ear was harvested and digested to get the cell suspension.

### Immunofluorescence staining

BMDMs or tissue sections were fixed with 4% paraformaldehyde in PBS or permeabilized with 100% methanol (chilled at -20°C) at room temperature for 5 min, blocked with 1% BSA in PBS for 30 min. Primary antibodies were incubated overnight at 4°C, followed by the secondary antibody for 1 hr at room temperature if needed or Hoechst 33342 for 5 min at room temperature. Antibodies used were FITC anti-mouse F4/80 antibody (BM8, 1:100, Biolegend), Alexa Fluor 647 anti-mouse CD86 antibody (GL-1, 1:100, Biolegend), Alexa Fluor 647 anti-mouse CD206 antibody (C068C2, 1:200, Biolegend), PE anti-mouse CD163 antibody (TNKUPJ, 1:40, eBioscience), anti-mouse arginase-1 antibody (24HCLC, 1:100, Invitrogen), Alexa Fluor 488 donkey anti-rabbit secondary antibody (A-21206, 1:1000, Invitrogen). Images were acquired using a Nikon A1MP+ fluorescence confocal microscope (Nikon, Japan) with a water-immersed 40× and 1.15 NA objective.

### Statistical Analysis

Data were evaluated with Graphpad Prism software (version 6.0) using unpaired two-tailed Student's t-tests. Data are expressed as means ± the standard deviation (SD). Differences were considered significant when p values were below 0.05.

## Results

### Mitochondria elongation and fusion might support the differentiation of monocytes toward macrophages

**Figure 1:**
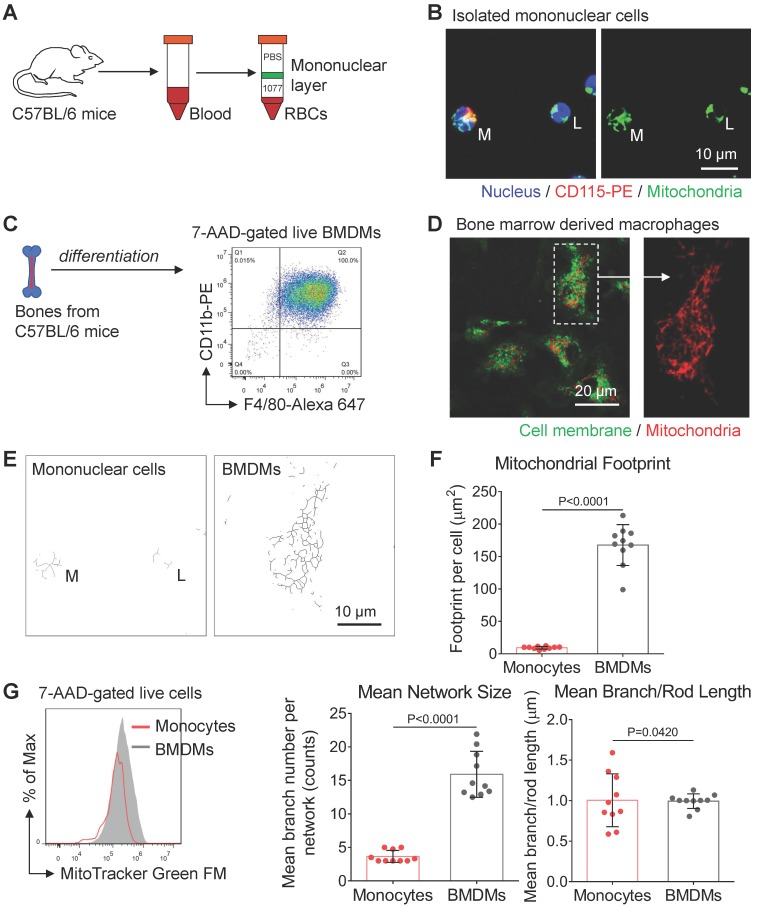
** Mitochondrial morphology varies from peripheral blood monocytes to bone marrow-derived macrophages (BMDMs). (A)** Schematic diagram showing the isolation of mononuclear leukocytes from the peripheral blood of mice using Histopaque (1.077 g/mL) banding method. **(B)** Fluorescence confocal images of mitochondria (green color, MitoTracker Green FM), nuclei (blue color, Hoechst 33342), and surface marker (red color, CD115-PE) in monocytes (M) and lymphocytes (L). Scale bar, 10 µm. **(C)** Schematic diagram showing the preparation of naive bone marrow-derived macrophages (BMDMs) using macrophage colony-stimulating factor (M-CSF) stimulation. The macrophage lineage was validated by the F4/80-Alexa 647 vs CD11b-PE scatter plot. **(D)** Fluorescence confocal images of mitochondria (red color, MitoTracker Red CMXRos) and cellular membrane (green color, PKH67 Green) in BMDMs. Scale bar, 20 µm. **(E)** Mitochondrial network morphology of mononuclear cells (corresponds to Figure 1B) and BMDMs (corresponds to Figure 1D) produced by ImageJ macro tools. Scale bar, 10 µm. **(F)** Mitochondrial organization analysis of monocytes and BMDMs. Mitochondrial footprint reflects mitochondrial mass in a cell, mean network size is the mean number of branches per network, while mean branch/rod length is the average size of all rods/branches. Dots are data from individual cells (n=10). **(G)** Flow cytometer data of MitoTracker Green FM fluorescence in monocytes (red line) and BMDMs (gray area). All data shown were representative of two or three independent runs of experiments.

Under challenging conditions, circulating blood monocytes will be recruited to inflammation sites and differentiate into macrophages 27. It is now becoming clear that monocytes reprogram their metabolism to facilitate monocyte-macrophage activation. However, relatively little is known about mitochondrial morpho-dynamics during monocyte-macrophage differentiation. As a preliminary examination, we used the histopaque to isolate mononuclear cells from mice peripheral blood (green band in Figure 1A). We found CD115-positive monocytes (M) had more tubular mitochondria (Figure 1B) than lymphocytes (L). These data agree with previous findings that mitochondria of peripheral-blood monocytes from a healthy donor are tubular and interconnected 28. Moreover, flow cytometer analysis showed that monocytes had more mitochondrial mass compared to lymphocytes (not shown in this paper). These data could also explain why monocytes have higher bioenergetic capability than that of lymphocytes at their basal state in previous studies 29.

To characterize the mitochondrial morphology after monocyte-macrophage differentiation, we used mouse bone marrow cells to develop BMDMs 30. Five days after treating macrophage colony-stimulating factor (M-CSF), high purity BMDMs were harvested and prepared for further examination (Figure 1C). After mitochondria staining, we found naive BMDMs had elongated and interconnected mitochondria (Figure 1D). To quantitatively evaluate mitochondrial structures, we used ImageJ macro tools to analyze mitochondrial network morphology 25. Compared with peripheral-blood monocytes, naive BMDMs had more mitochondrial mass, including mitochondrial footprints and network size (Figure 1E and 1F). These findings suggest that similar to stem cell differentiation 31, mitochondria of monocytes undergo biosynthesis and elongation during monocyte-macrophage differentiation (Figure 1E and 1G). Past research revealed that peripheral blood monocytes mainly fuel cell function by both glycolysis and OXPHOS at basal state 29, while the M-CSF induced BMDMs are featured by more active OXPHOS due to elevated expression of M2 macrophages-like genes 4, 32. Meanwhile, mitochondrial elongation elevates OXPHOS activity 18. Collectively, mitochondrial elongation and fusion might support more energetic requirements of OXPHOS during monocyte-macrophage differentiation.

### Mitochondrial morphology correlates with the metabolism-dependent activation of macrophages *in vitro*

**Figure 2:**
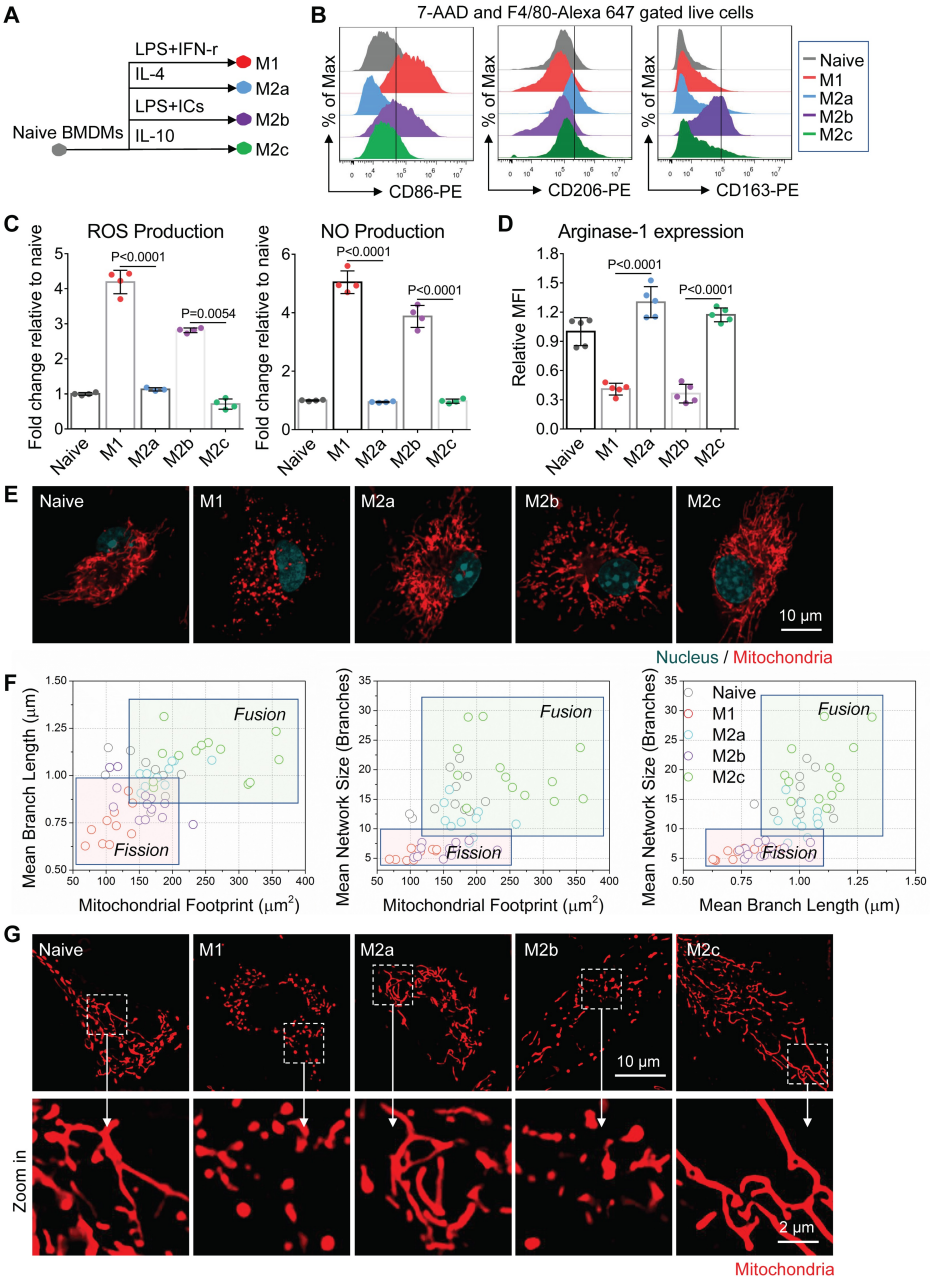
** Mitochondria organization correlates with the activation status of BMDMs *in vitro*. (A)** Schematic diagram showing the activation of bone marrow-derived macrophages (BMDMs) using different cytokines. Naive BMDMs were represented by gray color, M1 by red color, M2a by blue color, M2b by violet color, and M2c by green color. **(B)** After 24 hr of cytokine treatment, a flow cytometer was used to assess CD86, CD206, CD163 expression of BMDMs (naive, M1, M2a, M2b, and M2c). **(C)** After 24 hr of cytokine treatment, reactive oxygen species (ROS) and nitric oxide (NO) production of BMDMs were measured using DCFH-DA and DAF-FM DA fluorescent probes, respectively. **(D)** Flow cytometer was used to assess the intracellular arginase-1 expression of BMDMs. Fold changes of expression relative to naive were evaluated by mean fluorescence intensity (MFI). **(E)** Fluorescence confocal images of mitochondria (red color, MitoTracker Red CMXRos), and nuclei (blue color, Hoechst 33342) in BMDMs. Scale bar, 10 µm. **(F)** 2D scatter plots from mitochondrial network analysis (footprints, network size, and branch length) performed on BMDMs (naive, M1, M2a, M2b, and M2c). The quadrants of fission/fusion were added to enclose clustered macrophages in feature space based on their mitochondrial organization. The coordinates of each dot represent the feature values of individual cells (n≥10). (**G**) Representative structured illumination microscopy (SIM) images of mitochondria (red color, MitoTracker Red CMXRos) in BMDMs. Scale bar, 10 µm. All data shown were representative of two or three independent runs of experiments.

**Figure 3:**
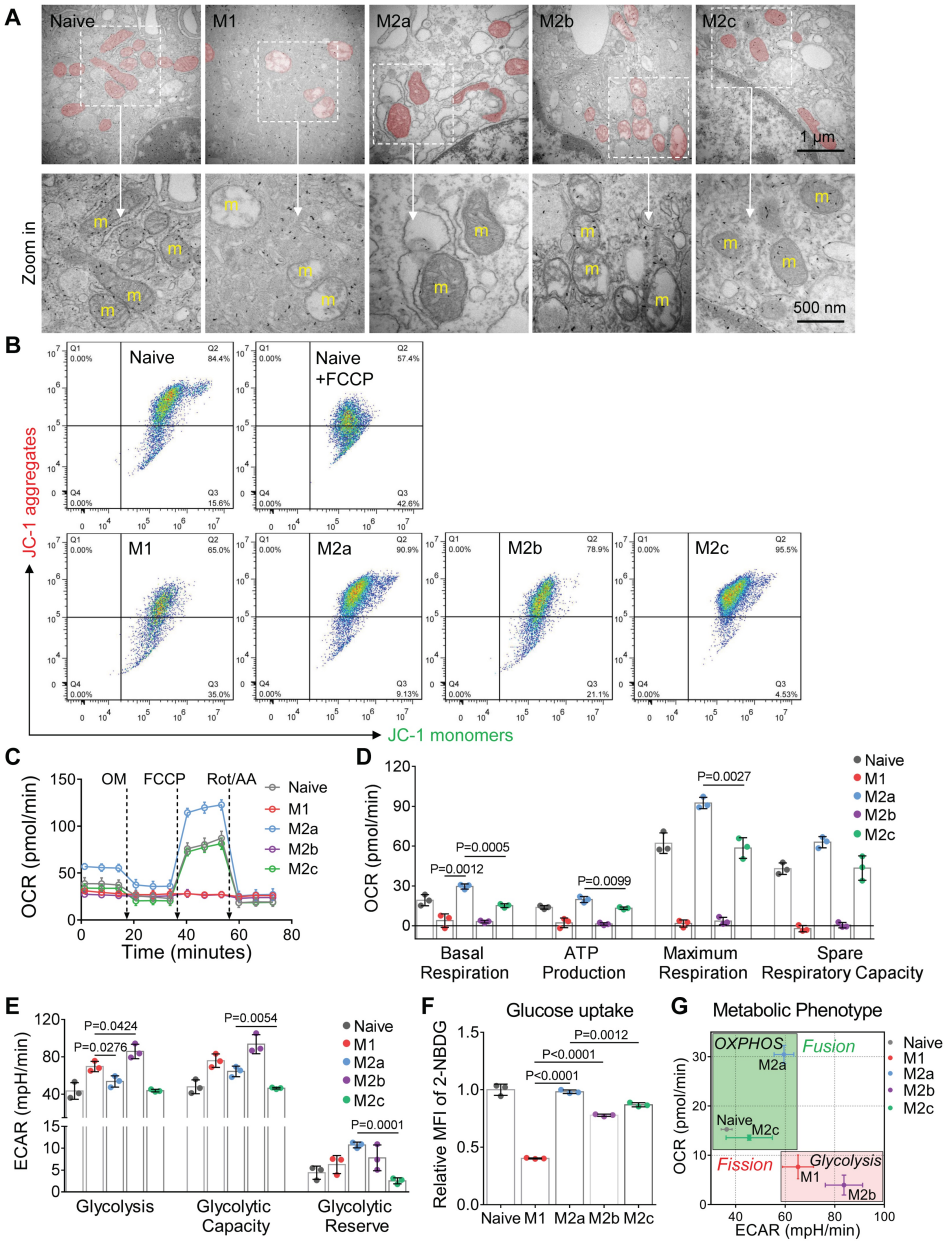
** Mitochondria regulate the cellular respiration of activated BMDMs *in vitro*. (A)** Representive transmission electron microscopy (TEM) images of mitochondria in BMDMs. Scale bar, 1 µm. **(B)** Mitochondrial membrane potential (ΔΨm) was measured using JC-1 probes. 100 μM FCCP-treated naive BMDMs as a positive control. A shifted population to lower fluorescence in the JC-1 aggregates channel shows the mitochondrial membrane potential is compromised. **(C)** The oxygen consumption rate (OCR) of BMDMs at baseline and in response to sequential treatment with oligomycin (OM), FCCP, and rotenone plus antimycin A (Rot/AA). **(D)** Basal respiration, ATP production, maximum respiration, and spare respiratory capacity of BMDMs at different activation status. **(E)** Glycolysis, glycolytic capability, and glycolytic reserve of BMDMs at different activation status. (**F**) Glucose uptake capability of BMDMs was measured using 2-NBDG fluorescent probes. Fold changes of 2-NBDG uptake were evaluated by MFI. (**G**) The mapping of metabolic phenotype based on the basal OCR and basal ECAR of BMDMs (naive, M1, M2a, M2b, and M2c). All data shown were representative of two or three independent runs of experiments.

Naive BMDMs can be further activated into two functionally polarized populations, termed as M1 (stimulated by IFN-γ [interferon γ] combined with LPS [lipopolysaccharide]) and M2 (M2a by IL-4 [interleukin 4]; M2b by immune-complexes combined with LPS; M2c by IL-10 [interleukin 10]) 33. These activated macrophages fuel featured functions by reprogramming cell metabolism. Metabolic profiles of M1 and M2a macrophages were well studied and defined in the past decade 34. Moreover, it is now becoming clear that fusion and fission directly control the function of mitochondria in cell respiration 20, 35. Together, it's reasonable to infer that the mitochondria organization will change with the activation of macrophages. Here, we developed four subtypes of activated macrophages from naive BMDMs, including M1, M2a, M2b, and M2c (illustrated in Figure 2A). Phenotyping on surface markers (Figure 2B) and functional assay (Figure 2C and 2D) showed that naive macrophages were successfully polarized into expected activation statuses. Using confocal microscopy, structured illumination microscopy (SIM) and transmission electron microscopy (TEM), we found that M1 and M2b macrophages had highly fragmented and discrete mitochondria, while M2a and M2c macrophages had elongated and interconnected ones (Figure 2E, 2G and 3A).

Among all the status of activation, the M1 macrophages had the least mitochondrial footprints, smallest network size, and shortest branch/rod length (Figure 2F, and S1B). These organization features might be due to excessive mitochondrial fission via de-phosphorylation of Drp1 36, 37. We further investigated the bioenergetic profiles of M1 macrophages by measuring the oxygen consumption rate (OCR) and extracellular acidification rate (ECAR). We found that OCR of M1 macrophages did not change with the serial administration of inhibitors (Figure 3C and 3D), indicating impaired OXPHOS capability of mitochondria 11. The TEM images showed that M1 macrophages had punctate and swell mitochondria with damaged cristae (Figure 3A). Moreover, the JC-1 assay revealed M1 macrophages typically had low mitochondrial membrane potential (Figure 3B). These findings verified the dysfunction of mitochondria in M1 macrophages. Under such conditions, M1 macrophages can only exploit glycolysis to fuel ATP requirements and thus elevated their lactate production (Figure 3E). Recent evidences revealed that lactate might maintain the glycolysis state of M1 macrophages by lactylation of histone lysine residues 38. Besides, the truncated TCA cycle indirectly elevates glycolysis flux through PPP, while active PPP provides a large amount of NADPH for NADPH oxidase-mediated ROS and NO generation 15. Our results confirmed that M1 macrophages had higher ROS and NO production (Figure 2C), and much lower Arginase-1 expression (Figure 2D).

Similarly, mitochondria of M2b macrophages also have small network sizes, less mitochondrial footprints, and short mean branch/rod length (Figure 2F and S1B). Due to the stimulation of LPS, M2b macrophages also featured by the low arginase-1 expression, high iNOS activity, high ROS production, and impaired OXPHOS (Figure 2C, 2D and 3C) 36, 37, 39. The major difference between M1 and M2b are the expression of CD163 (Figure 2B). However, different from pro-inflammatory M1 macrophages, M2b macrophages possess both immune-regulated and anti-inflammatory functions 40. They have more mitochondrial footprints than M1 (Figure 2F and S1B), which might result from the elevated level of IL-10 41. Recent studies show that IL-10 has anti-inflammatory effects by eliminating dysfunctional mitochondria 42. JC-1 assay revealed that M1 macrophages had more impaired mitochondria than that of M2b macrophages (Figure 3B). Moreover, the functional assay also showed that M2b macrophages had lower NO and ROS production than M1 macrophages (Figure 2C). Such a clearance mechanism will reduce the proportion of punctate organization induced by LPS. Moreover, we found that M2b macrophages had a higher acidification rate, spare respiratory capacity, and glycolysis capacity compared to M1 macrophages (Figure 3D and 3E). This feature of M2b might result from a relatively higher glucose uptake of M2b (Figure 3F).

In contrast to M1 and M2b, pro-healing M2a macrophages had more interconnection among mitochondria (Figure 2E, and 2G). Their organizations have significantly higher values in footprints, network size, and branch length (Figure 2F and S1B). As mentioned before, M-CSF induced BMDMs were M2-like macrophages and had fused mitochondria. We found that M2a macrophages had higher OXPHOS than naive BMDMs, including basal respiration, maximum respiration, and spare respiration capability (Figure 3D), possibly caused by these following reasons. Firstly, IL-4 elevates the expression of OXPHOS-related genes in M2a macrophages to support the enhanced OXPHOS 43. Moreover, IL-4 was proved to promote mitochondrial fusion in macrophages 44. TEM showed that M2a macrophages had more tubular mitochondria with dense and folded cristae (Figure 3A). Mitochondrial fusion can improve the efficiency of mitochondrial electron transport chain (ETC) by increasing cristae formation and supercomplexes formation, further maximizing OXPHOS capability 17, 45. Last, M2a macrophages also fuel energy by FAO in addition to glucose metabolism 46, while fused mitochondria can facilitate the utilization of fatty acids via trafficking lipid droplets 47. Functionally, we found that M2a macrophages markedly elevated the expression of Arginase-1 (Figure 2D), its resulting polyamines are important for wound healing. Moreover, elevated Arginase-1 can block iNOS activity by the competitive combination of its substrate (arginine) and down-regulated NO generation, which is consistent with our findings (Figure 2C). Together, fused mitochondria elevate the efficient operation of OXPHOS in M2a macrophages, further support their anti-inflammatory and wound healing function.

M2c macrophages had larger interconnected mitochondria similar to M2a macrophages (Figure 2E and 2F). Interestingly, M2c macrophages had even fewer punctate mitochondria (Figure 2E and 2G) and lower ROS production (Figure 2C) than naive BMDMs, possibly caused by that IL-10 can eliminate dysfunctional mitochondria via mitophagy 42. JC-1 assay revealed M2c macrophages had a much lower percentage of dysfunctional mitochondria relative to others (Figure 3B). However, in contrast to what we expected, we found that M2c macrophages had slightly decreased basal respiration and glycolytic reserve relative to naive BMDMs (Figure 3D and 3E). Previous studies pointed out that IL-10 eases glucose uptake by regulating the glucose transporter (GLUT1) translocation, and inhibits glycolysis by downregulating the gene expression of glycolytic enzymes 42. Our glucose uptake assay (Figure 3F) further supports this notion. Therefore, the reduction of glucose uptake might be a reason accounting for the reduced basal respiration and glycolytic reserve.

Overall, the metabolic phenotype of OCR and ECAR can effectively differentiate the activation status of macrophages (Figure 3G). The metabolism reprogramming during activation was highly dependent on the mitochondrial organization. These three quantitative parameters of footprints, network size, and branch length can extract the organization features of macrophage mitochondria. In the corresponding two-dimension (2D) scatter plots (Figure 2F), the data points of naive and four major activation status were clustered and separated from each other. These results indicate that morpho-phenotypes of mitochondria can serve as a metabolic index to characterize the activation status of macrophages in the contexts.

### Imaging mitochondria helps to dissect macrophage activation status *in situ*

**Figure 4:**
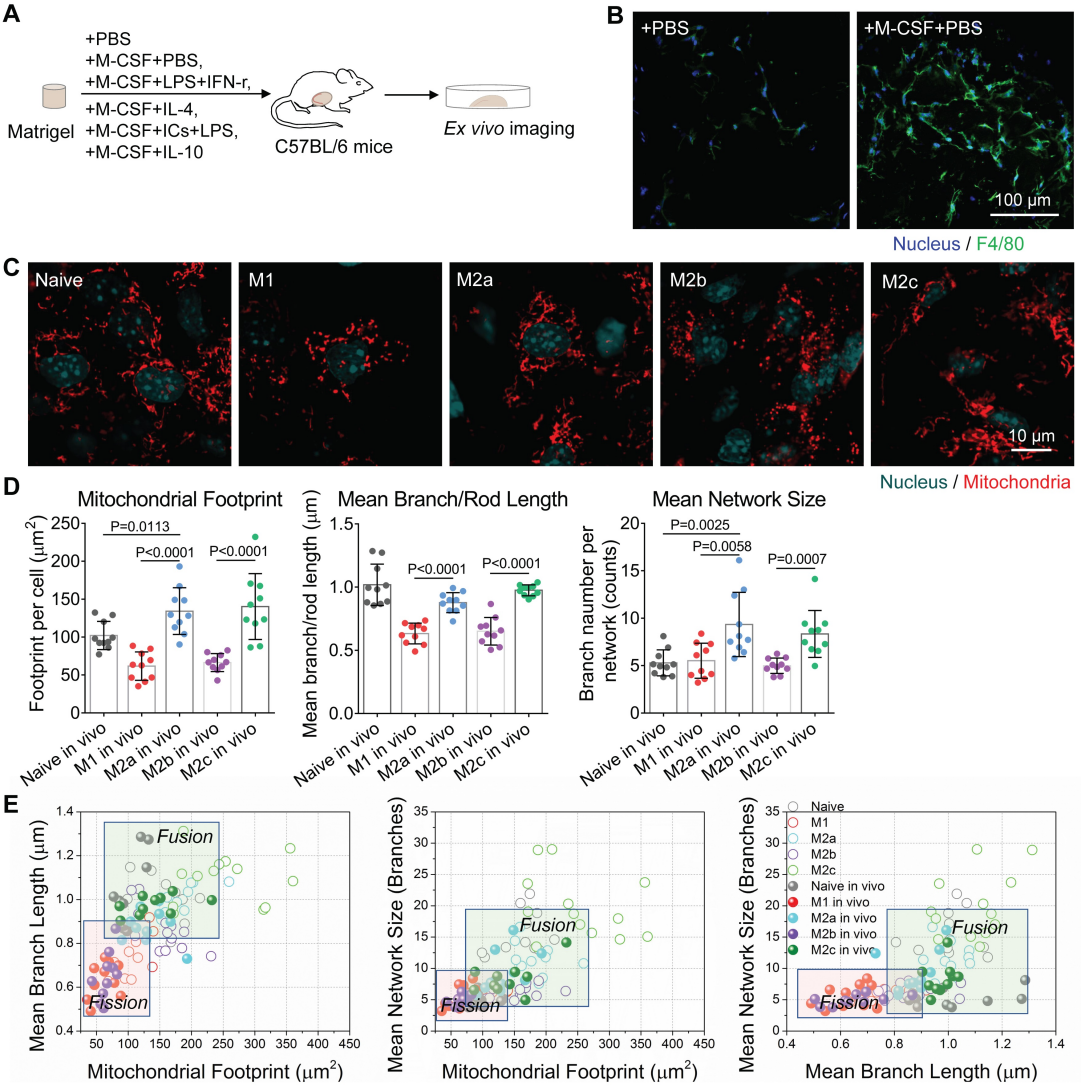
** Mitochondrial dynamics reflects the activation status of Matrigel plug-recruited macrophages *in situ*. (A)** Schematic overview of experiment procedures, including Matrigel mixture preparation, subcutaneous injection and *ex vivo* imaging on Matrigel plugs. To generated activated macrophages in mice, M-CSF+PBS were used for naive macrophage differentiation, M-CSF+LPS+IFN-γ for M1, M-CSF+IL-4 for M2a, M-CSF+immune-complexes+LPS for M2b, M-CSF+IL-10 for M2c, and PBS only for the control group. **(B)** Immunofluorescence imaging of F4/80 expression (green) of fixed cells in implanted Matrigel plugs. The nuclei are stained with Hoechst 33342 (blue). Scale bar, 100 µm. **(C)** *Ex vivo* live-cell imaging on mitochondria (red color, MitoTracker Red CMXRos), and nuclei (blue color, Hoechst 33342) in different types of macrophages recruited by Matrigel plugs (naive, M1, M2a, M2b, and M2c). Scale bar, 10 µm. **(D)** Quantitative analysis of mitochondrial organization on macrophages from Matrigel plugs (footprint, network size, and branch length). Dots are data from individual cells (n≥10). **(E)** Comparison of 2D scatter plots from mitochondrial organization parameters between BMDMs (Naive, M1, M2a, M2b, and M2c) *in vitro* and macrophages of Matrigel plugs (Naive *in vivo*, M1* in vivo*, M2a* in vivo*, M2b* in vivo*, M2c* in vivo*). The quadrants of fission/fusion were added to enclose clustered macrophages in feature space based on their mitochondrial organization. Dots are data of individual cells (n≥10). All data shown were representative of two or three independent runs of experiments.

Physical and mechanical transductions may regulate the activation and phenotypes of macrophages 48. Compared to the 2D culture on a plastic petri dish, softer Matrigel, a basement membrane matrix, provides a 3D growth environment with mechanical properties similar to those *in vivo* 49. To investigate whether the differences of mitochondrial morphology among different activation status are still significant *in situ*, we added M-CSF and various stimulating cytokines into growth factor-reduced Matrigel mixtures and subcutaneously implanted them into mice. Then the Matrigel plug attracted circulating monocytes and educated macrophages in mice (illustrated in Figure 4A). To avoid the effect of hypoxia and starvation on macrophage activation, the injection volume of Matrigel plugs were below 250 µL. On the 5^th^-day post-implantation, large numbers of F4/80 positive macrophages (Figure 4B right panel, green color) infiltrated Matrigel plugs. In contrast, the phosphate-buffered saline (PBS) control group attracts quite a few (Figure 4B left panel). Evaluated by the intensities of immunofluorescence, the relative expression levels of surface markers (CD86, CD206, and CD163) and functional enzymes (Arginase-1) in these F4/80 positive macrophages (Figure S2A) agreed with the designed activation. These data confirmed that macrophages derived from peripheral blood monocytes were successfully activated in Matrigel plug just like those from BMDMs.

To characterize the mitochondrial organization in these activated macrophages, we took out Matrigel plugs from mice and incubated them with a completed medium for *ex vivo* imaging. Morphologically, we found that M1 and M2b macrophages were still featured by punctate mitochondria, and M2a and M2c macrophages were featured by interconnected mitochondria (Figure 4C). Quantitatively, OXPHOS-active M2a and M2c macrophages still had bigger mitochondrial footprints, a larger networks, and longer branches compared to glycolytic M1 and M2b macrophages (Figure 4D, and S2B). M2c has a longer mean branch length than M2a's (Figure 4D). All the cell types have decreased mitochondria footprints (solid circles in Figure 4E). This shrinking of organization parameters might be due to a 2D projection of 3D networks. The soft environment may play some roles to affect the proportion of shrinkage, which is different among activation types. Even so, the organization features of glycolytic M1 and M2b macrophages are still separable from those of OXPHOS supported M2a and M2c ones (Figure 4E).

### Mitochondria provides a metabolic index to monitor M2a macrophage repolarization

**Figure 5:**
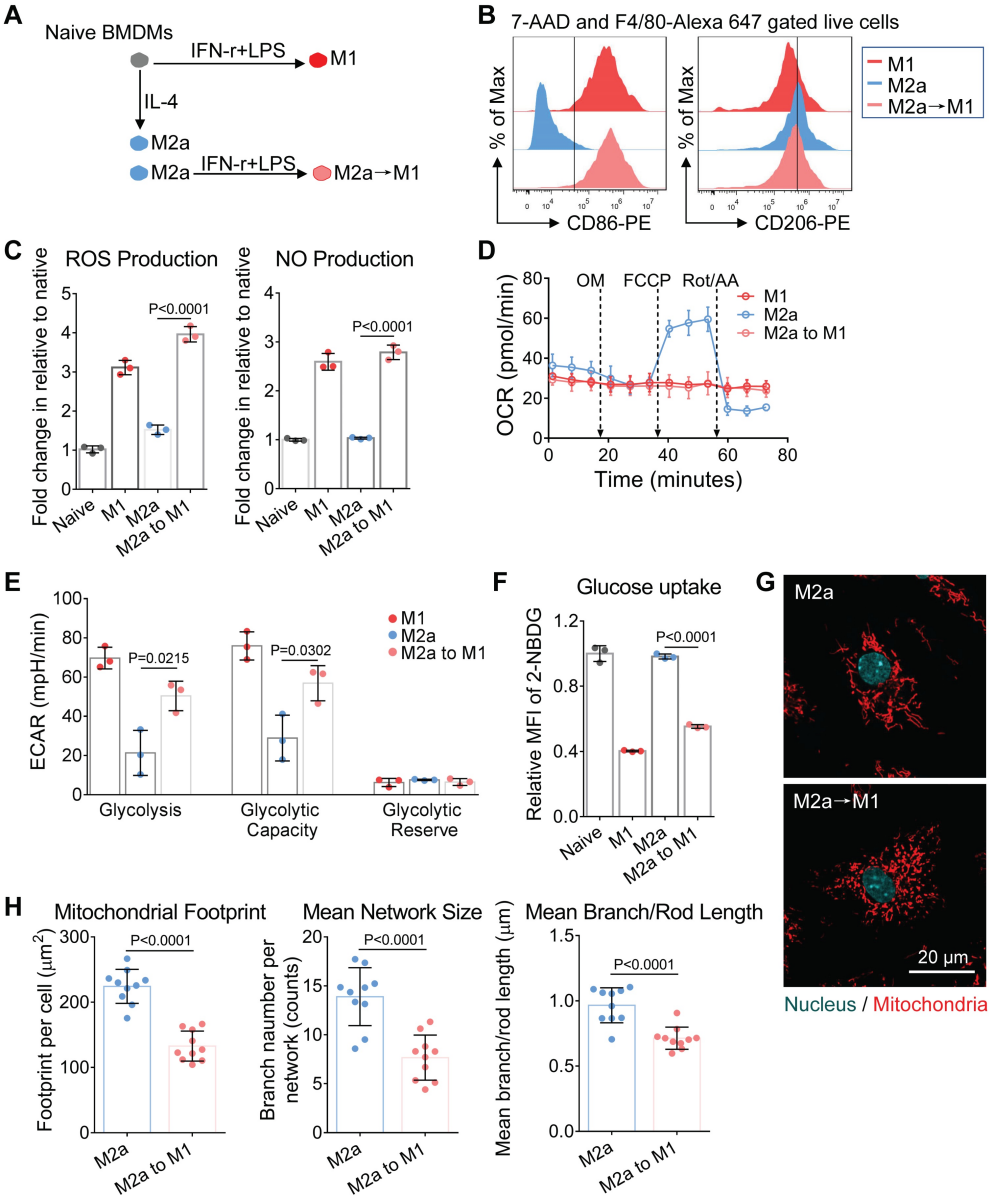
** Mitochondrial morphology changes along with the repolarization of M2a macrophages. (A)** Schematic illustration of the M1-activation of anti-inflammatory M2a macrophages. BMDMs were primed with IL-4 for 24 hr to generate M2a macrophages; then cells were treated with IFN-γ plus LPS for an additional 24 hr to generate M2a→M1 macrophages. **(B)** Flow cytometer data of CD86-PE and CD206-PE expression in M1, M2a, and M2a→M1 macrophages. **(C)** The ROS and NO production levels in naive, M1, M2a, and M2a→M1macrophages, measured by DCFH-DA and DAF-FM DA fluorescent probes, respectively.** (D)** Changes of OCR in M1, M2a, and M2a→M1 macrophages with intervention. **(E)** Changes of glycolysis capability in M1, M2a, and M2a→M1 macrophages with intervention. **(F)** Glucose uptake capabilities of naive, M1, M2a, and M2a→M1 macrophages were measured by 2-NBDG fluorescent probes. **(G)** Fluorescence confocal images of mitochondria (red color, MitoTracker Red CMXRos), and nuclei (blue color, Hoechst 33342) in M2a and M2a→M1 macrophages. Scale bar, 20 µm. **(H)** Changes of the mitochondrial organization during the repolarization of M2a macrophages (M2a, M2a→M1). n=10. All data shown were representative of two or three independent runs of experiments.

Editing the disease-promoting macrophages into opposite activation status may bring therapeutic effects. For instance, the switch of M2-like TAMs to inflammatory M1 macrophages 50-53 has been considered as a treatment strategy against cancer 54. However, it is challenging to non-invasively monitor the dynamic change of macrophage activation status in the course of treatment. In the present study, to track the organization dynamics of macrophage mitochondria during manipulation, we further performed an M2a to M1 re-activation by stimulating M2a macrophages with LPS+IFN-γ (Figure 5A). Interestingly, we found that the treatment of LPS+IFN-γ elevated the expression of inflammatory surface marker (CD86) but didn't remove M2a macrophage's surface marker (CD206) on M2a→M1 cells (Figure 5B). Functionally, M2a→M1 cells had elevated NO and ROS production, which was even higher than those of M1 macrophages (Figure 5C).

We found that similar to M1 macrophages, the OCR of M2a→M1 cells did not change after the serial administration of several inhibitors (Figure 5D). The M2a→M1 cells also exhibited elevated glycolysis (Figure 5E) and impaired glucose uptake (Figure 5F). Additionally, we found that M1 and M2a→M1 cells had similar punctate mitochondria (Figure 5G and 5H), indicating that LPS+IFN-r treatment induced mitochondrial fission during M1-activation. These data validated that mitochondrial fragmentation occurs when anti-inflammatory macrophages are activated to a pro-inflammatory phenotype. However, M2a→M1 cells still retained their CD206 surface markers (Figure 5B), which might mislead the identification of macrophage activation if only one marker was evaluated. In this situation, the mitochondria organization could serve as a helpful index to reveal the treatment response of macrophages in the contexts such as tumor microenvironment.

### NO maintains M1 macrophage activation by blocking the mitochondrial dynamics

Conversely, editing pro-inflammatory M1 macrophages toward pro-healing M2 macrophages seems a very attractive way to mitigate the inflammation in diseases such as obesity, chronic wounds, and atherosclerosis 10, 55, 56. However, M2-activation of M1 macrophages remains a challenge, mainly caused by their mitochondrial dysfunction 11. The reasons why the mitochondria of M1 macrophages could not restore M2 metabolic functions even after the removal of inflammatory conditions remain unanswered. Based on the fragmentation dynamics of mitochondria organization in M2a→M1 macrophages, we hypothesized that the fusion of mitochondria may be required for the M1→M2 repolarization.

**Figure 6:**
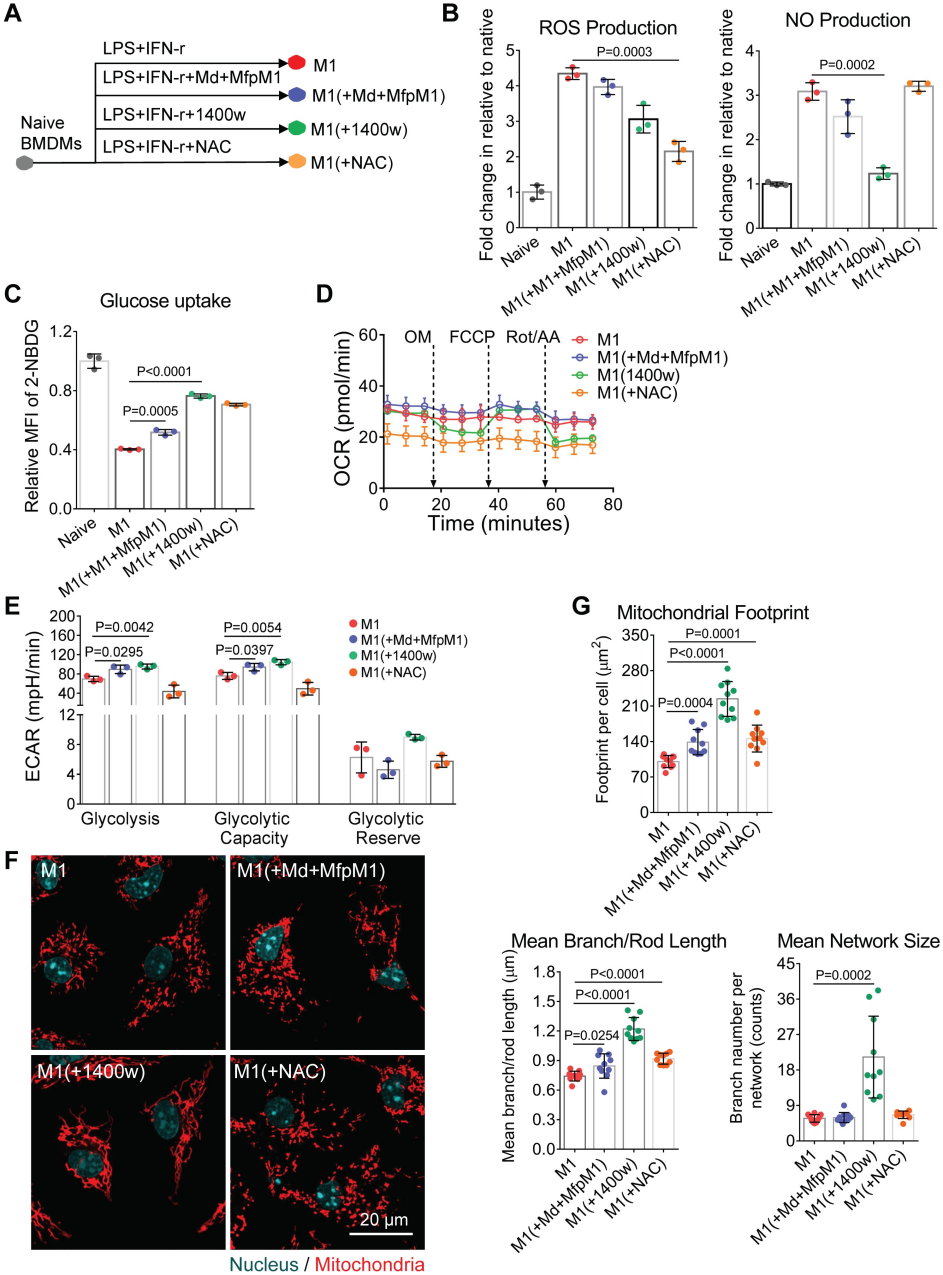
** NO blocks the mitochondrial fusion of M1 macrophages. (A)** Schematic illustration of the intervention on M1 macrophage activation. The BMDMs were stimulated for 24 hr with IFN-γ plus LPS either in the presence or absence of Mdivi-1 (mitochondrial fission inhibitor) plus MfpM1 (mitochondrial fusion promoter), 1400W (the inhibitor of iNOS), or NAC (N-acetylcysteine, the ROS scavenger). **(B)** ROS production and NO production of macrophages were measured using DCFH-DA and DAF-FM DA fluorescent probes respectively. **(C)** Glucose uptake capability of macrophages (M1, M1[+Mdivi-1+MfpM1], M1[+1400w], and M1[+NAC]) were measured using 2-NBDG fluorescent probes. **(D)** Changes of OCR during M1 polarization with intervention (control, [+Md+MfpM1], [+1400w], and [+NAC]). **(E)** Changes of glycolytic capability during M1 polarization with intervention (control, [+Md+MfpM1], [+1400w], and [+NAC]). **(F)** Mitochondrial morphology of macrophages (M1, M1[+Mdivi-1+MfpM1], M1[+1400w], and M1[+NAC]). Mitochondria are red (MitoTracker Red CMXRos), and nuclei are cyan (Hoechst 33342). Scale bar, 20 µm. **(G)** Changes of mitochondrial organization during M1 polarization with intervention (control, [+Md+MfpM1], [+1400w], and [+NAC]). n=10. All data shown were representative of two or three independent runs of experiments.

To examine this hypothesis, we treated macrophages with Mdivi-1 (mitochondrial fission inhibitor) plus mitochondrial fusion promoter M1 (MfpM1) during LPS + IFN-r treatment (Figure 6A). A combination of Mdivi-1 and MfpM1 has been proved to restore the mitochondrial function of glycolytic effector T cells via promoting mitochondrial fusion 20. In this study, Mdivi-1+MfpM1 failed to change the expression of the M1 surface marker (Figure S3A) and didn't ameliorate mitochondria fission in M1 macrophages (Figure 6F and 6G). Although we did not observe increased OXPHOS in Mdivi-1+MfpM1 treated cells (Figure 6D and S3B), they elevated metabolic activity by increasing glycolysis capability (Figure 6E and S3C), which was consistent with the findings in T cell experiments 20. Increasing glycolysis of M1(+Mdivi-1+MfpM1) cells might be supported by elevated glucose uptake capability (Figure 6C). Mitochondrial fusion/fission is mainly regulated by the ratio of fusion proteins (Mfn1) to fission proteins (Drp1) 57. Baker and colleagues found that LPS induces mitochondrial fission in macrophages via the de-phosphorylation and activation of Drp1 and the degradation of Mfn1 in an interleukin 1 receptor-associated kinase (IRAK-1)-dependent fashion 37. Therefore, LPS-induced unbalance of Mfn1-to-Drp1 ratio might be one of the reasons leading to the failure of Mdivi-1+MfpM1 to edit the metabolic function in M1 macrophages.

Moreover, accumulating evidence suggest that ROS and NO could regulate mitochondrial morphology and function via chemical reaction 58. Katoh and colleagues showed that mitochondrial ROS production contributes to mitochondrial fission in microglia under inflammatory conditions 59. To test the effect of ROS or NO on the reserve of mitochondrial function, we treated macrophages with NAC (N-acetylcysteine, the ROS scavenger), and 1400w (the inhibitor of iNOS) during LPS + IFN-r stimulation (Figure 6A). We found that NAC markedly inhibited ROS production in M1 macrophages (Figure 6B). However, similar to Mdivi-1+MfpM1, NAC also failed to block the expression of inflammatory surface marker (Figure S3A) and mitochondrial fission (Figure 6F and 6G), and to retain M1 mitochondrial function (Figure 6D and S3B). These findings indicate that there are additional triggers, rather than ROS, regulating mitochondrial dynamics in M1 macrophages.

NO was demonstrated to induce mitochondrial fission in neurons 60. Interestingly, we found only 1400w improved metabolic function of mitochondria in M1 macrophages, including basal respiration, ATP production and maximum respiration (Figure 6D and S3B). Moreover, 1400w also markedly elevated the glucose uptake capability of M1(+1400w) cells in relative to M1 macrophages (Figure 6C). Morphologically, we found that 1400w markedly blocked the mitochondrial fragmentation in M1 macrophages (Figure 6F), which were consistent with the findings from metabolomics assay. Aside from retaining OXPHOS capability, 1400w also elevated glycolysis in M1 macrophages (Figure 6E and S3C), which might be supported by elevated glucose uptake (Figure 6C). Functionally, Mdivi-1+MfpM1 and NAC failed to block the activity of iNOS whereas 1400w inhibited NO production in M1 macrophages (Figure 6B). Previous studies indicate that the genetical deletion of iNOS was proved to improve mitochondrial function in M1 macrophages 11. Moreover, NO triggers persistent mitochondrial fission via S-nitrosylation of Drp1, and the S-nitrosylation is an irreversible protein modification process 61, 62, highlighting that NO production might be another reason leading to the failure of Mdivi-1+MfpM1 on promoting fusion. Collectively, these findings may explain why M1 macrophages could not be easily repolarized to M2 activation. Our method provides metabolic insights more than surface markers did.

### Preparation and characterization of MitoTracker-loaded liposomes for macrophage mitochondria labeling

**Figure 7:**
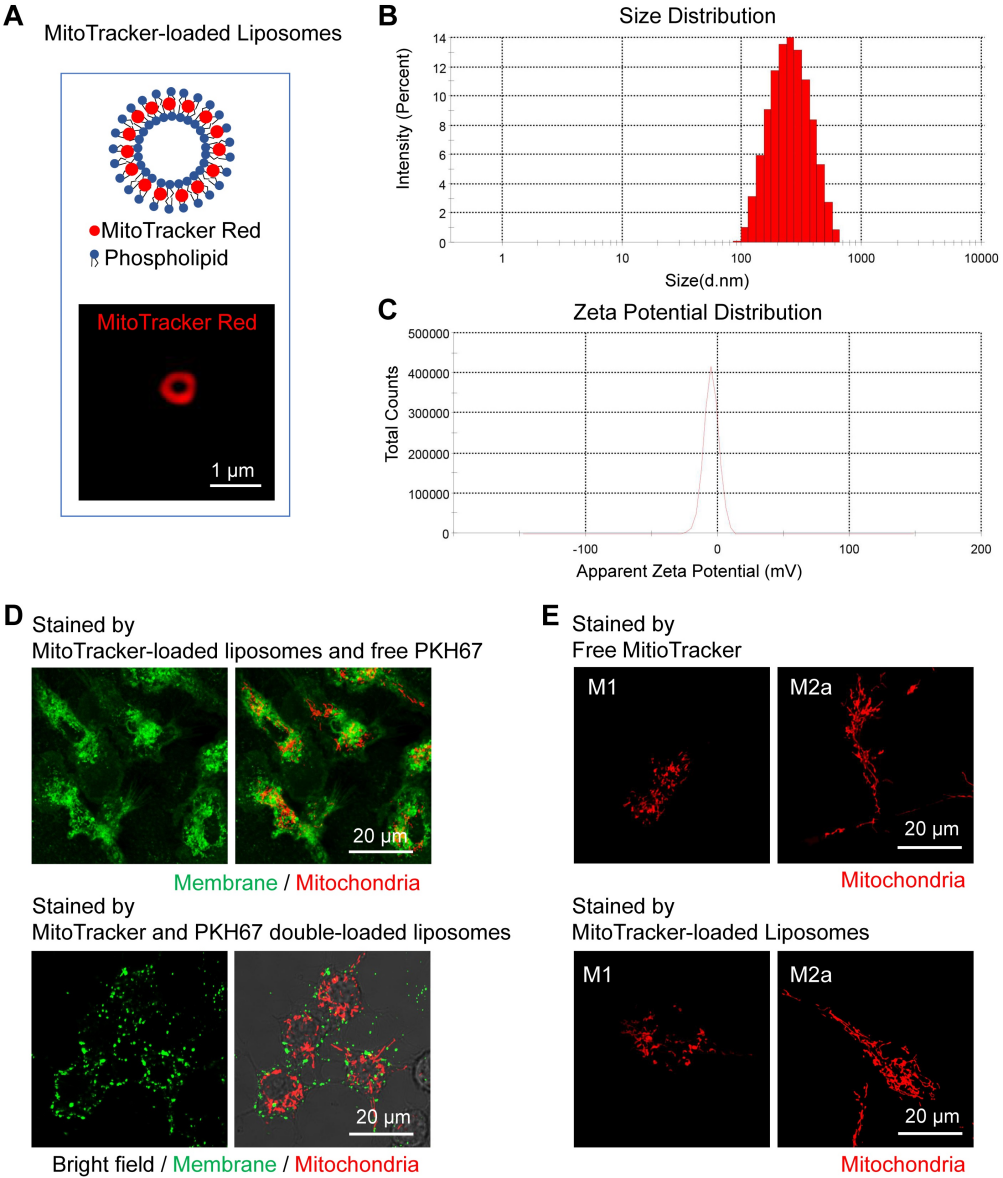
** Characterization of MitoTracker-loaded liposomes *in vitro*. (A)** Schematic structure of MitoTracker-loaded liposome and the representative fluorescent image of liposome (red color, MitoTracker Red CMXRos) captured by SIM. Scale bar, 1 µm. **(B-C)** The distribution of sizes and surface zeta potentials of MitoTracker-loaded liposomes diluted by water.** (D)** The fluorescence confocal images of labeled membrane and mitochondria in naive BMDMs from C57BL/6 mice. Two ways of labeling, MitoTracker-loaded liposomes subsequently with free PKH67 dye (upper panel) and MitoTracker\PKH67 double-loaded liposomes simultaneously (lower panel), were compared. Cell morphology (gray, bright field), mitochondria (red color, MitoTracker Red CMXRos) and membrane structures (green color, PKH67 Green). Scale bar, 20 µm. **(E)** The fluorescence confocal images of labeled mitochondria in BMDMs (M1 and M2a activation) from C57BL/6 mice. Two ways of labeling, free MitoTracker, and MitoTracker-loaded liposomes, were compared. Mitochondria (red color, MitoTracker Red CMXRos). Scale bar, 20 µm. All data shown were representative of two or three independent runs of experiments.

Even though there are already various *in vitro* methods to label mitochondrial structures 63, it's still challenging to *in vivo* visualize mitochondrial morphology and dynamics in targeted cells. Clodronate-contained liposomes have been proven its efficacy and specificity to deplete macrophages in organs and tissues 64. Besides, fluorescent dyes-loaded liposomes have been used to label and track macrophages *in vivo* 65. Inspired by these approaches, here we developed MitoTracker-loaded liposomes to label the mitochondria of macrophages *in vivo* selectively. As shown in the 561 nm excited confocal images (Figure 7A), the hydrophobic MitoTracker Red probes were loaded on the lipid bilayers of liposomes. The mean diameter of the liposomes was around 200 nm (Figure 7B), and the mean zeta potential was about -5.9 mV when dissolved in water (Figure 7C). Both 1120 nm and 1180 nm wavelengths can effectively excite the two-photon red fluorescence of the MitoTracker dyes (Figure S4A). The 617 nm peak wavelength of two-photon fluorescence spectra in liposomes (blue-solid and blue-dashed curves in Figure S4A) were longer than the 607 nm peaks of free MitoTracker d yes in PBS solution 66, and mitochondria bounded MitoTracker (red-solid and deep red-dashed curves in Figure S4A).

To confirm the labeling efficacy, we firstly administered the MitoTracker-loaded liposomes to the activated BMDMs *in vitro.* After the engulfment by cells, the MitoTracker dyes successfully released from the MitoTracker-loaded liposomes and labeled the mitochondria in BMDMs (Figure 7D, upper panel). MitoTracker and PKH67 double-loaded liposomes failed to label the cell membrane (Figure 7D, lower panel) compared to free PKH67 dye staining (Figure 7D, upper panel), indicating that the MitoTracker was intracellularly released from intact liposomes after endocytosis. Moreover, the process of liposome uptake didn't cause obvious alterations on the mitochondrial morphology of activated (M1 and M2a) macrophages (Figure 7E).

### Imaging mitochondria *in vivo* reveals the metabolic transition of macrophages in the course of inflammation

**Figure 8:**
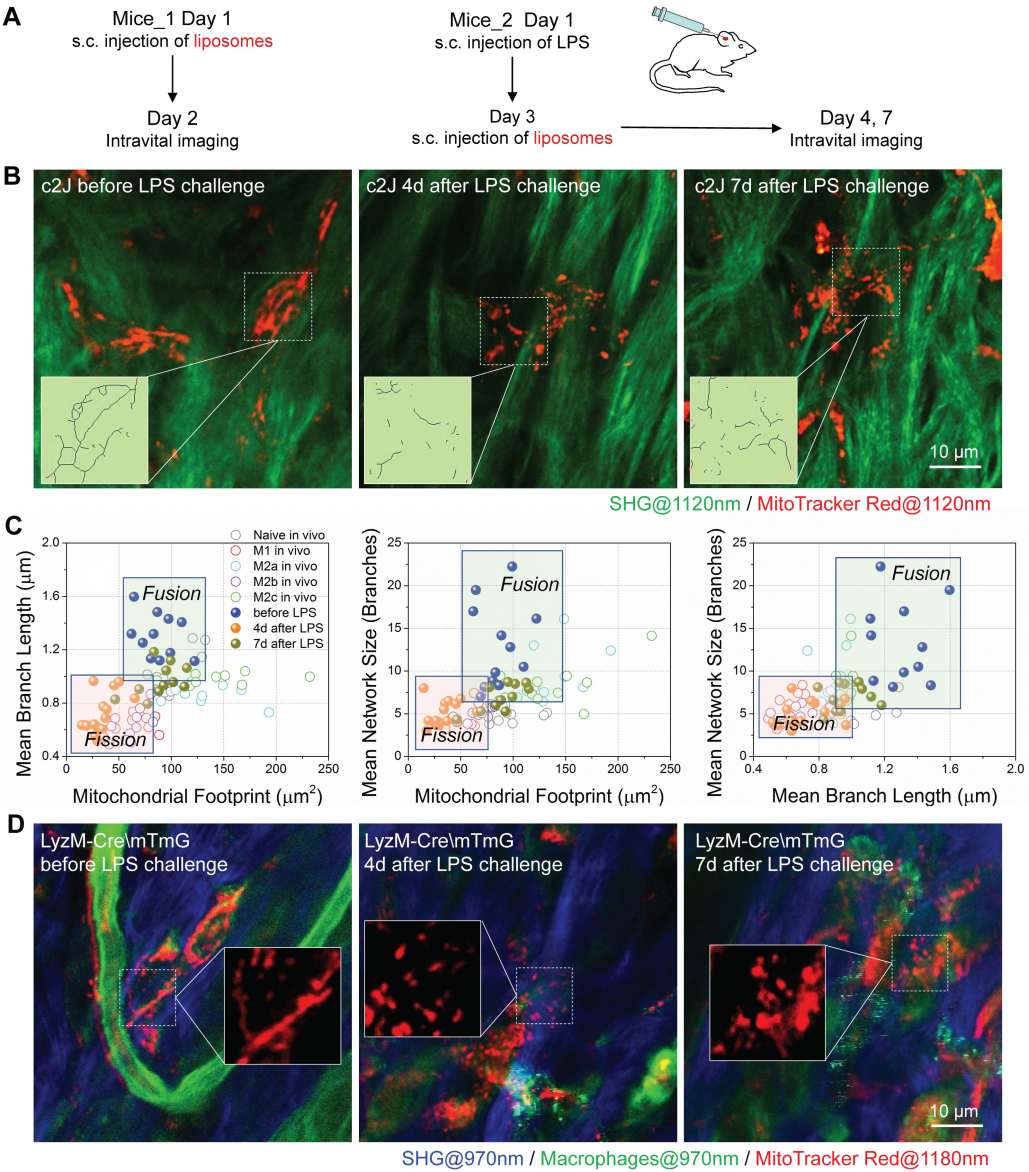
** MitoTracker-loaded liposomes target mitochondria of macrophages in mice. (A)** Experimental protocol. (1) To observe the mitochondria of resident macrophages, MitoTracker-loaded liposomes were subcutaneously injected into the dorsal side of mice ear pinna one day before *in vivo* imaging. (2) The LPS was subcutaneously injected into the dorsal side of mice ear pinna to prime inflammation. On the day 3 after LPS injection, MitoTracker-loaded liposomes were subcutaneously injected into the LPS-challenged mice ear. On the day 4 and day 7 after LPS injection, mice were anesthetized for two-photon *in vivo* imaging. **(B)** Second harmonic generation (SHG, green) images of collagens and two-photon fluorescence images of mitochondria (red) in c2J mice ear before/after LPS challenge. The excitation wavelength was 1120 nm. Scale bar, 10 µm. **(C)** 2D scatter plots of mitochondrial organization parameters in macrophages from Matrigel plugs (Naive *in vivo*, M1* in vivo*, M2a* in vivo*, M2b* in vivo*, and M2c* in vivo*) and c2J mice ear (before LPS, 4d after LPS, and 7d after LPS). The quadrants of fission/fusion were added to enclose clustered macrophages in feature space based on their mitochondrial organization. Dots are data of individual cells (n≥10). **(D)** Second harmonic generation (SHG) images of collagens (blue; excited at 970 nm) and two-photon fluorescence images of macrophage mitochondria (red; excited at 1180 nm) in LysM-Cre\mTmG mice ear before/after LPS challenge. Scale bar, 10 µm. All data shown were representative of two or three independent runs of experiments.

For the *in vivo* delivery test, we subcutaneously injected the MitoTracker-loaded liposomes into the ear of c2J mice (procedures illustrated in Figure 8A). One day after liposome injection, we used a multiphoton microscope excited at 1120 nm to observe the sectioning images *in vivo* and found some red fluorescence-labeled cells distributing in mice ear. The 607 nm fluorescent peaks of these labeled cells (Figure S4B) were consistent with those of free or mitochondria bounded MitoTracker Red (Figure S4A), which means the labeling dyes have left the liposome and labeled mitochondria *in vivo*. The immunofluorescence staining of mice ears harvested before, and on the 4^th^-day after LPS challenge indicated that MitoTracker-liposome labeled cells were positive for F4/80 antibody staining (Figure S5). Data above validated that subcutaneously administered MitoTracker-loaded liposomes can be selectively delivered to macrophages and stained their mitochondria *in vivo*. These resident macrophages showed connected and elongated mitochondria in the dermis layer of the normal ear (Figure 8B left panel, and more detail provided in Figure S6).

To observe the mitochondrial dynamics after inflammatory stimulation, we challenged the ear of c2J mice via subcutaneous LPS injection 67. Flow cytometer and immunofluorescence imaging (Figure S4C and S4D) results of excised tissues revealed that resident macrophages expressed higher M2a/M2c markers (CD206) and lower M1/M2b markers (CD86), indicating that resident macrophages might be M2a/M2c-like macrophages before LPS challenge. As we expected, the LPS treatment elicited M1/M2b-like activation of macrophages. After the LPS challenge, abundant F4/80 positive macrophages infiltrated the inflammatory ear (Figure S4D), and they had higher CD86 expression and lower CD206/CD163 expression (Figure S4C and S4D), indicating newly recruited macrophages from peripheral blood were activated to M1/M2b-like phenotype under LPS treatment. On the 7^th^-day after LPS challenge, these F4/80 positive macrophages had a higher level of M2a/M2c marker expression but they also retained high expression of M1/M2b marker, revealing that this cell population still contain some M1/M2b macrophages (Figure S4C and S4D).

MitoTracker-loaded liposomes were subcutaneously injected into LPS-challenged mice ear three days after the LPS challenge (Figure 8A). On the 4^th^-day post LPS challenge, we found the mitochondria were discrete and fragmented (Figure 8B middle panel, and Figure S6). They showed more connection again on the 7^th^-day post LPS challenge (Figure 8B right panel, and Figure S6). By extracting the backbones of mitochondria networks (Figure 8B), we analyzed their organization parameters, projected these data points in the 2D and 3D scatter plots, and compared them with those from Matrigel plugs (Figure 8C and S4E). As we expected, the organization features of resident macrophages distributed in the zone of OXPHOS preferred activation status (naive, M2a, and M2c). After the LPS challenge, the organization feature points of macrophages *in vivo* separated from resident ones and clustered to the left-bottom region in all 2D plots, where the glycolytic M1 and M2b macrophages reside (Figure 8C). After the resolution of the LPS challenge, on the 7^th^-day post LPS challenge, the distribution of data points moved back to the region where OXPHOS-preferred macrophages populated (Figure 8C). These findings were consistent with the results of phenotyping on surface markers (Figure S4C and S4D).

To verify the specificity of liposome delivery *in vivo*, we employed a C57BL/6 transgenic (LysM-Cre\ mTmG) mice in which LysM-Cre positive macrophages would express the enhanced green fluorescence proteins (EGFP) on the cellular membranes. For other cell types that lack the expression of lysozyme will express tdTomato fluorescence proteins instead. The BMDMs of LysM-Cre\mTmG mice have the EGFP expression (Figure S7A), which verified the turn-on of EGFP expression in lysozyme positive macrophages. We used 970 nm femtosecond pulses to efficiently excite two-photon fluorescence of EGFP (green color in Figure S7B). According to the literature, the two-photon absorption cross-section of tdTomato almost vanished at 1180 nm 68. Therefore, we excited the two-photon fluorescence of MitoTracker Red at 1180 nm (red color in Figure S7B) to avoid bleed-through contamination from tdTmoato. With such dual-color excited two-photon microscopy in transgenic mice, we found that the 1180 nm excited two-photon red fluorescence (red color in Figure S7B) can be observed in EGFP positive cells. The corresponding 607 nm fluorescence peak in each cell (Figure S7B) verified the presence of MitoTracker dyes (Figure S4A). Similar to the results of c2J mice, before the LPS challenge, macrophages had elongated tubular mitochondria, whereas EGFP positive macrophages in LPS-challenged ear had discrete and punctate mitochondria on the 4^th^-day post LPS injection (Figure 8D). Since most neutrophils will disappear on the 3^rd^-day post LPS induction, this colocalization of probes and LysM^+^ cells indicate the success of *in vivo* MitoTracker delivery to macrophages in mice ear. Together, our results proved that MitoTracker-labeled liposomes could be used to selectively label macrophages and observe their mitochondria *in vivo*. Thus obtained organization features of mitochondria can reflect the metabolic dynamics of macrophages and differentiate their activation types into M1/M2b and naive/M2a/M2c categories.

## Discussion

In this work, we developed a method to characterize the metabolic phenotype and activation status of activated macrophages *in vivo*, and further to speculate their functions in the complicated and dynamic immune microenvironment. To verify the conclusions obtained by confocal microscopy, we also used super-resolution microscopy to examine the structures of mitochondria in BMDMs. The results of N-SIM and TEM prove that our conclusions obtained in confocal images are valid and reliable. However, the tissues need to be fixed and sectioned before the TEM observation. It's not possible for TEM to characterize the *in vivo* activation status and dynamics of macrophages. Although the SIM reveals better structural differences (transverse resolution ~120 nm) *in vitro*, considering the imaging depth and *in vivo* applicability, we still need to adopt confocal and multiphoton microscopy to locate and analyze the mitochondria organization *in situ* and *in vivo*. At first glance, it's a little bit difficult to distinguish the differences of mitochondria morphology in these confocal images. With the help of quantitative morphology analysis, we successfully extracted the organization features of mitochondria in confocal or multiphoton images and further enhanced their differences. This approach has translational potential for clinical diagnosis, especially for these chronic wounds and mucosal inflammation diseases. Moreover, subcellular imaging of mitochondria helps to track the transition of macrophage activation *in vitro* and *in vivo* even when the surface markers are ambiguous for identification, which provides a visual index for the evaluation of macrophage-targeted therapeutics.

On the way toward the method development and testing, we also found many interesting features of mitochondria in macrophages. First, the monocytes expanded their mitochondrial networks to facilitate cell function during monocyte-macrophage differentiation. However, accumulating studies demonstrate that blood monocytes with abnormal mitochondria are strongly correlated with systemic inflammatory diseases 28, 69, 70. Moreover, increased expression of iNOS was found in circulating monocytes from patients with inflammatory diseases 71. Based on these reports and our results, we speculate that pro-inflammatory cytokines in peripheral blood might suppress mitochondrial expansion via elevating NO production in circulating monocytes, leading to abnormal monocyte differentiation. These findings highlight that healthy mitochondria in peripheral monocytes are important for proper monocyte activation and the mitochondria organization of monocytes may act as a diagnostic marker for systemic inflammatory diseases. Second, we found that NO-induced mitochondrial fragmentation leads to their dysfunction in inflammatory macrophages, which can be markedly suppressed by the iNOS inhibitor. These results enlighten us that iNOS may serve as a therapeutic target to control inflammatory diseases and promote the repolarization of M1 macrophages.

Although the TEM, transgenic strategy and mitochondria-specific dyes help to make mitochondria visible, label-free methods are still the preferable tools to achieve the least perturbation to their metabolism and functions. NADH, a metabolic fluorophore, presents in the cytoplasm and mitochondria of cells in free or protein-bound status. Free NADH is mainly localized in the cytosol, while bound NADH is predominately localized in the mitochondria. Imaging on NADH can reflect mitochondrial boundary in live cells because NADH fluorescence is predominantly derived from bound NADH 72, 73. Recently, Dimitra Pouli and colleagues developed an approach to monitor mitochondrial dynamics in human skin by imaging NADH with two-photon fluorescence microscopy. They successfully differentiated malignant lesions from healthy skin epithelia based on the NADH revealed mitochondrial patterns 74. However, this method can't selectively image macrophages and their mitochondrial dynamics due to limited specificity. Fortunately, with continuing progress in optical imaging techniques, third-harmonic generation (THG) imaging based on granularity differences and three-photon autofluorescence (3PAF) imaging based on NADH differences have been used to achieve the identification of macrophages without any label 75, 76. Combined imaging techniques will help to identify macrophages and their activation status via label-free imaging simultaneously in the near future. Collectively, we believe these findings will advance the study of various inflammation-related diseases, such as cancer, diabetes mellitus, autoimmune diseases, and chronic wounds.

## Supplementary Material

Supplementary figures.

## Abbreviations

BMDMs: bone marrow derived macrophages
Drp1: dynamin-related protein 1
ECAR: extracellular acidification rate
EGFP: enhanced green fluorescent protein
ETC: electron transport chain
FAO: fatty acid oxidation
FCCP: fluoro-carbonyl cyanide phenylhydrazone
FLIM: fluorescence lifetime imaging microscopy
GLUT-1: glucose transporter 1
IFN-γ: interferon γ
IL-10: interleukin 10
IL-4: interleukin 4
iNOS: inducible nitric oxide synthase
IRAK-1: interleukin 1 receptor-associated kinase
LPS: lipopolysaccharide
M-CSF: macrophage colony-stimulating factor
Mdivi-1: mitochondrial mivision inhibitor 1
Mfn1: mitofusin 1
MfpM1: mitochondrial fusion promoter M1
NAC: N-acetylcysteine
NO: nitric oxide
OCR: oxygen consumption rate
OM: oligomycin
Opa-1: optic atrophy 1
OXPHOS: oxidative phosphorylation
PBS: phosphate buffered saline
PPP: pentose phosphate pathway
RBCs: red blood cells
ROS: reactive oxygen species
SHG: second harmonic generation
SIM: structured illumination microscopy
TAMs: tumor-associated macrophages
TEM: transmission electron microscopy
1400w: dihydrochloride
2D: two dimensional
2-NBDG: 2-(N-(7-Nitrobenz-2-oxa-1,3-diazol-4-yl)Amino)-2-Deoxyglucose
3D: three dimensional

## References

[1] Martinez FO, Gordon S (2014). The M1 and M2 paradigm of macrophage activation: time for reassessment. F1000Prime Rep.

[2] Pollard JW (2009). Trophic macrophages in development and disease. Nat Rev Immunol.

[3] O'Neill LA, Pearce EJ (2016). Immunometabolism governs dendritic cell and macrophage function. J Exp Med.

[4] Van den Bossche J, Baardman J, de Winther MP (2015). Metabolic Characterization of Polarized M1 and M2 Bone Marrow-derived Macrophages Using Real-time Extracellular Flux Analysis. J Vis Exp.

[5] Szulczewski JM, Inman DR, Entenberg D, Ponik SM, Aguirre-Ghiso J, Castracane J (2016). In Vivo Visualization of Stromal Macrophages via label-free FLIM-based metabolite imaging. Sci Rep.

[6] Sriram R, Nguyen J, Santos JD, Nguyen L, Sun J, Vigneron S (2018). Molecular detection of inflammation in cell models using hyperpolarized (13)C-pyruvate. Theranostics.

[7] Wu C, Yue X, Lang L, Kiesewetter DO, Li F, Zhu Z (2014). Longitudinal PET imaging of muscular inflammation using 18F-DPA-714 and 18F-Alfatide II and differentiation with tumors. Theranostics.

[8] Geeraerts X, Bolli E, Fendt SM, Van Ginderachter JA (2017). Macrophage Metabolism As Therapeutic Target for Cancer, Atherosclerosis, and Obesity. Front Immunol.

[9] Chen D, Xie J, Fiskesund R, Dong W, Liang X, Lv J (2018). Chloroquine modulates antitumor immune response by resetting tumor-associated macrophages toward M1 phenotype. Nat Commun.

[10] Liu YC, Zou XB, Chai YF, Yao YM (2014). Macrophage polarization in inflammatory diseases. Int J Biol Sci.

[11] Van den Bossche J, Baardman J, Otto NA, van der Velden S, Neele AE, van den Berg SM (2016). Mitochondrial Dysfunction Prevents Repolarization of Inflammatory Macrophages. Cell Rep.

[12] Archer SL (2013). Mitochondrial dynamics-mitochondrial fission and fusion in human diseases. N Engl J Med.

[13] van der Bliek AM, Shen Q, Kawajiri S (2013). Mechanisms of mitochondrial fission and fusion. Cold Spring Harb Perspect Biol.

[14] Jheng HF, Tsai PJ, Guo SM, Kuo LH, Chang CS, Su IJ (2012). Mitochondrial fission contributes to mitochondrial dysfunction and insulin resistance in skeletal muscle. Mol Cell Biol.

[15] Yu T, Robotham JL, Yoon Y (2006). Increased production of reactive oxygen species in hyperglycemic conditions requires dynamic change of mitochondrial morphology. Proc Natl Acad Sci U S A.

[16] Li JY, Zhang K, Xu D, Zhou WT, Fang WQ, Wan YY (2018). Mitochondrial Fission Is Required for Blue Light-Induced Apoptosis and Mitophagy in Retinal Neuronal R28 Cells. Front Mol Neurosci.

[17] Cogliati S, Frezza C, Soriano ME, Varanita T, Quintana-Cabrera R, Corrado M (2013). Mitochondrial cristae shape determines respiratory chain supercomplexes assembly and respiratory efficiency. Cell.

[18] Yao CH, Wang R, Wang Y, Kung CP, Weber JD, Patti GJ (2019). Mitochondrial fusion supports increased oxidative phosphorylation during cell proliferation. Elife.

[19] Gomes LC, Di Benedetto G, Scorrano L (2011). During autophagy mitochondria elongate, are spared from degradation and sustain cell viability. Nat Cell Biol.

[20] Buck MD, O'Sullivan D, Klein Geltink RI, Curtis JD, Chang CH, Sanin DE (2016). Mitochondrial Dynamics Controls T Cell Fate through Metabolic Programming. Cell.

[21] Wang Y, Subramanian M, Yurdagul A Jr, Barbosa-Lorenzi VC, Cai B, de Juan-Sanz J (2017). Mitochondrial Fission Promotes the Continued Clearance of Apoptotic Cells by Macrophages. Cell.

[22] Garaude J, Acin-Perez R, Martinez-Cano S, Enamorado M, Ugolini M, Nistal-Villan E (2016). Mitochondrial respiratory-chain adaptations in macrophages contribute to antibacterial host defense. Nat Immunol.

[23] Ying W, Cheruku PS, Bazer FW, Safe SH, Zhou B (2013). Investigation of macrophage polarization using bone marrow derived macrophages. J Vis Exp.

[24] Broggi A, Cigni C, Zanoni I, Granucci F (2016). Preparation of Single-cell Suspensions for Cytofluorimetric Analysis from Different Mouse Skin Regions.

[25] Valente AJ, Maddalena LA, Robb EL, Moradi F, Stuart JA (2017). A simple ImageJ macro tool for analyzing mitochondrial network morphology in mammalian cell culture. Acta Histochem.

[26] Jaafar-Maalej C, Diab R, Andrieu V, Elaissari A, Fessi H (2010). Ethanol injection method for hydrophilic and lipophilic drug-loaded liposome preparation. J Liposome Res.

[27] Sieweke MH, Allen JE (2013). Beyond stem cells: self-renewal of differentiated macrophages. Science.

[28] Widlansky ME, Wang J, Shenouda SM, Hagen TM, Smith AR, Kizhakekuttu TJ (2010). Altered mitochondrial membrane potential, mass, and morphology in the mononuclear cells of humans with type 2 diabetes. Transl Res.

[29] Chacko BK, Kramer PA, Ravi S, Johnson MS, Hardy RW, Ballinger SW (2013). Methods for defining distinct bioenergetic profiles in platelets, lymphocytes, monocytes, and neutrophils, and the oxidative burst from human blood. Lab Invest.

[30] Murray PJ, Allen JE, Biswas SK, Fisher EA, Gilroy DW, Goerdt S (2014). Macrophage activation and polarization: nomenclature and experimental guidelines. Immunity.

[31] Fang D, Yan S, Yu Q, Chen D, Yan SS (2016). Mfn2 is Required for Mitochondrial Development and Synapse Formation in Human Induced Pluripotent Stem Cells/hiPSC Derived Cortical Neurons. Sci Rep.

[32] Na YR, Hong JH, Lee MY, Jung JH, Jung D, Kim YW (2015). Proteomic Analysis Reveals Distinct Metabolic Differences Between Granulocyte-Macrophage Colony Stimulating Factor (GM-CSF) and Macrophage Colony Stimulating Factor (M-CSF) Grown Macrophages Derived from Murine Bone Marrow Cells. Mol Cell Proteomics.

[33] Mantovani A, Sica A, Sozzani S, Allavena P, Vecchi A, Locati M (2004). The chemokine system in diverse forms of macrophage activation and polarization. Trends Immunol.

[34] Langston PK, Shibata M, Horng T (2017). Metabolism Supports Macrophage Activation. Front Immunol.

[35] Mishra P, Chan DC (2016). Metabolic regulation of mitochondrial dynamics. J Cell Biol.

[36] Park J, Choi H, Kim B, Chae U, Lee DG, Lee SR (2016). Peroxiredoxin 5 (Prx5) decreases LPS-induced microglial activation through regulation of Ca(2+)/calcineurin-Drp1-dependent mitochondrial fission. Free Radic Biol Med.

[37] Baker B, Maitra U, Geng S, Li L (2014). Molecular and cellular mechanisms responsible for cellular stress and low-grade inflammation induced by a super-low dose of endotoxin. J Biol Chem.

[38] Zhang D, Tang Z, Huang H, Zhou G, Cui C, Weng Y (2019). Metabolic regulation of gene expression by histone lactylation. Nature.

[39] Xu H, Sun L, He Y, Yuan X, Niu J, Su J (2019). Deficiency in IL-33/ST2 Axis Reshapes Mitochondrial Metabolism in Lipopolysaccharide-Stimulated Macrophages. Front Immunol.

[40] Wang LX, Zhang SX, Wu HJ, Rong XL, Guo J (2019). M2b macrophage polarization and its roles in diseases. J Leukoc Biol.

[41] Edwards JP, Zhang X, Frauwirth KA, Mosser DM (2006). Biochemical and functional characterization of three activated macrophage populations. J Leukoc Biol.

[42] Ip WKE, Hoshi N, Shouval DS, Snapper S, Medzhitov R (2017). Anti-inflammatory effect of IL-10 mediated by metabolic reprogramming of macrophages. Science.

[43] Mills EL, O'Neill LA (2016). Reprogramming mitochondrial metabolism in macrophages as an anti-inflammatory signal. Eur J Immunol.

[44] Gao Z, Li Y, Wang F, Huang T, Fan K, Zhang Y (2017). Mitochondrial dynamics controls anti-tumour innate immunity by regulating CHIP-IRF1 axis stability. Nat Commun.

[45] Mishra P, Carelli V, Manfredi G, Chan DC (2014). Proteolytic cleavage of Opa1 stimulates mitochondrial inner membrane fusion and couples fusion to oxidative phosphorylation. Cell Metab.

[46] Huang SC, Everts B, Ivanova Y, O'Sullivan D, Nascimento M, Smith AM (2014). Cell-intrinsic lysosomal lipolysis is essential for alternative activation of macrophages. Nat Immunol.

[47] Rambold AS, Cohen S, Lippincott-Schwartz J (2015). Fatty acid trafficking in starved cells: regulation by lipid droplet lipolysis, autophagy, and mitochondrial fusion dynamics. Dev Cell.

[48] McWhorter FY, Davis CT, Liu WF (2015). Physical and mechanical regulation of macrophage phenotype and function. Cell Mol Life Sci.

[49] Tigges U, Hyer EG, Scharf J, Stallcup WB (2008). FGF2-dependent neovascularization of subcutaneous Matrigel plugs is initiated by bone marrow-derived pericytes and macrophages. Development.

[50] Hagemann T, Lawrence T, McNeish I, Charles KA, Kulbe H, Thompson RG (2008). "Re-educating" tumor-associated macrophages by targeting NF-kappaB. J Exp Med.

[51] Guo L, Zhang Y, Wei R, Wang C, Feng M (2019). Lipopolysaccharide-anchored macrophages hijack tumor microtube networks for selective drug transport and augmentation of antitumor effects in orthotopic lung cancer. Theranostics.

[52] Zhao J, Zhang Z, Xue Y, Wang G, Cheng Y, Pan Y (2018). Anti-tumor macrophages activated by ferumoxytol combined or surface-functionalized with the TLR3 agonist poly (I: C) promote melanoma regression. Theranostics.

[53] Jin H, He Y, Zhao P, Hu Y, Tao J, Chen J (2019). Targeting lipid metabolism to overcome EMT-associated drug resistance via integrin beta3/FAK pathway and tumor-associated macrophage repolarization using legumain-activatable delivery. Theranostics.

[54] Poh AR, Ernst M (2018). Targeting Macrophages in Cancer: From Bench to Bedside. Front Oncol.

[55] Zhang S, Liu Y, Zhang X, Zhu D, Qi X, Cao X (2018). Prostaglandin E2 hydrogel improves cutaneous wound healing via M2 macrophages polarization. Theranostics.

[56] Choi JY, Ryu J, Kim HJ, Song JW, Jeon JH, Lee DH (2018). Therapeutic Effects of Targeted PPAR Activation on Inflamed High-Risk Plaques Assessed by Serial Optical Imaging In Vivo. Theranostics.

[57] Takamura H, Koyama Y, Matsuzaki S, Yamada K, Hattori T, Miyata S (2012). TRAP1 controls mitochondrial fusion/fission balance through Drp1 and Mff expression. PLoS One.

[58] Willems PH, Rossignol R, Dieteren CE, Murphy MP, Koopman WJ (2015). Redox Homeostasis and Mitochondrial Dynamics. Cell Metab.

[59] Katoh M, Wu B, Nguyen HB, Thai TQ, Yamasaki R, Lu H (2017). Polymorphic regulation of mitochondrial fission and fusion modifies phenotypes of microglia in neuroinflammation. Sci Rep.

[60] Barsoum MJ, Yuan H, Gerencser AA, Liot G, Kushnareva Y, Graber S (2006). Nitric oxide-induced mitochondrial fission is regulated by dynamin-related GTPases in neurons. EMBO J.

[61] Cho DH, Nakamura T, Fang J, Cieplak P, Godzik A, Gu Z (2009). S-nitrosylation of Drp1 mediates beta-amyloid-related mitochondrial fission and neuronal injury. Science.

[62] Cho B, Choi SY, Cho HM, Kim HJ, Sun W (2013). Physiological and pathological significance of dynamin-related protein 1 (drp1)-dependent mitochondrial fission in the nervous system. Exp Neurobiol.

[63] Harwig MC, Viana MP, Egner JM, Harwig JJ, Widlansky ME, Rafelski SM (2018). Methods for imaging mammalian mitochondrial morphology: A prospective on MitoGraph. Anal Biochem.

[64] Kelly C, Jefferies C, Cryan SA (2011). Targeted liposomal drug delivery to monocytes and macrophages. J Drug Deliv.

[65] Arwert EN, Harney AS, Entenberg D, Wang Y, Sahai E, Pollard JW (2018). A Unidirectional Transition from Migratory to Perivascular Macrophage Is Required for Tumor Cell Intravasation. Cell Rep.

[66] Bestvater F, Spiess E, Stobrawa G, Hacker M, Feurer T, Porwol T (2002). Two-photon fluorescence absorption and emission spectra of dyes relevant for cell imaging. J Microsc.

[67] Kataru RP, Jung K, Jang C, Yang H, Schwendener RA, Baik JE (2009). Critical role of CD11b+ macrophages and VEGF in inflammatory lymphangiogenesis, antigen clearance, and inflammation resolution. Blood.

[68] Drobizhev M, Makarov NS, Tillo SE, Hughes TE, Rebane A (2011). Two-photon absorption properties of fluorescent proteins. Nat Methods.

[69] Weiss SL, Selak MA, Tuluc F, Perales Villarroel J, Nadkarni VM, Deutschman CS (2015). Mitochondrial dysfunction in peripheral blood mononuclear cells in pediatric septic shock. Pediatr Crit Care Med.

[70] Bhansali S, Bhansali A, Walia R, Saikia UN, Dhawan V (2017). Alterations in Mitochondrial Oxidative Stress and Mitophagy in Subjects with Prediabetes and Type 2 Diabetes Mellitus. Front Endocrinol (Lausanne).

[71] Dijkstra G, Zandvoort AJ, Kobold AC, de Jager-Krikken A, Heeringa P, van Goor H (2002). Increased expression of inducible nitric oxide synthase in circulating monocytes from patients with active inflammatory bowel disease. Scand J Gastroenterol.

[72] Dumas JF, Argaud L, Cottet-Rousselle C, Vial G, Gonzalez C, Detaille D (2009). Effect of transient and permanent permeability transition pore opening on NAD(P)H localization in intact cells. J Biol Chem.

[73] Blinova K, Levine RL, Boja ES, Griffiths GL, Shi ZD, Ruddy B (2008). Mitochondrial NADH fluorescence is enhanced by complex I binding. Biochemistry-Us.

[74] Pouli D, Balu M, Alonzo CA, Liu Z, Quinn KP, Rius-Diaz F (2016). Imaging mitochondrial dynamics in human skin reveals depth-dependent hypoxia and malignant potential for diagnosis. Sci Transl Med.

[75] Tsai CK, Chen YS, Wu PC, Hsieh TY, Liu HW, Yeh CY (2012). Imaging granularity of leukocytes with third harmonic generation microscopy. Biomed Opt Express.

[76] You S, Tu H, Chaney EJ, Sun Y, Zhao Y, Bower AJ (2018). Intravital imaging by simultaneous label-free autofluorescence-multiharmonic microscopy. Nat Commun.

