# Supplementary material for: Imaging of macrophage mitochondria dynamics in vivo reveals cellular activation phenotype for diagnosis
Source: Theranostics. 2020 Feb 3;10(7):2897–917. doi: 10.7150/thno.40495 (PMC7053213; doi:10.7150/thno.40495)
Supplement: Supplementary file 1 — Supplementary figures. [file thnov10p2897s1.pdf]

Supplementary Materials

Imaging of macrophage mitochondria dynamics in vivo reveals cellular activation phenotype for diagnosis

Yue Li[1], Yuan He[2], Kai Miao[1], Ying Zheng[2], Chuxia Deng[1], Tzu-Ming Liu[1*]

[1]Institute of Translational Medicine, Faculty of Health Sciences, University of Macau, Taipa, Macao SAR, China

[2]Institute of Chinese Medical Sciences, University of Macau, Taipa, Macao SAR, China

*** Correspondence:** Tzu-Ming Liu. E-mail address:tmliu@umac.mo

Supplementary figures 1-7

Figure S1

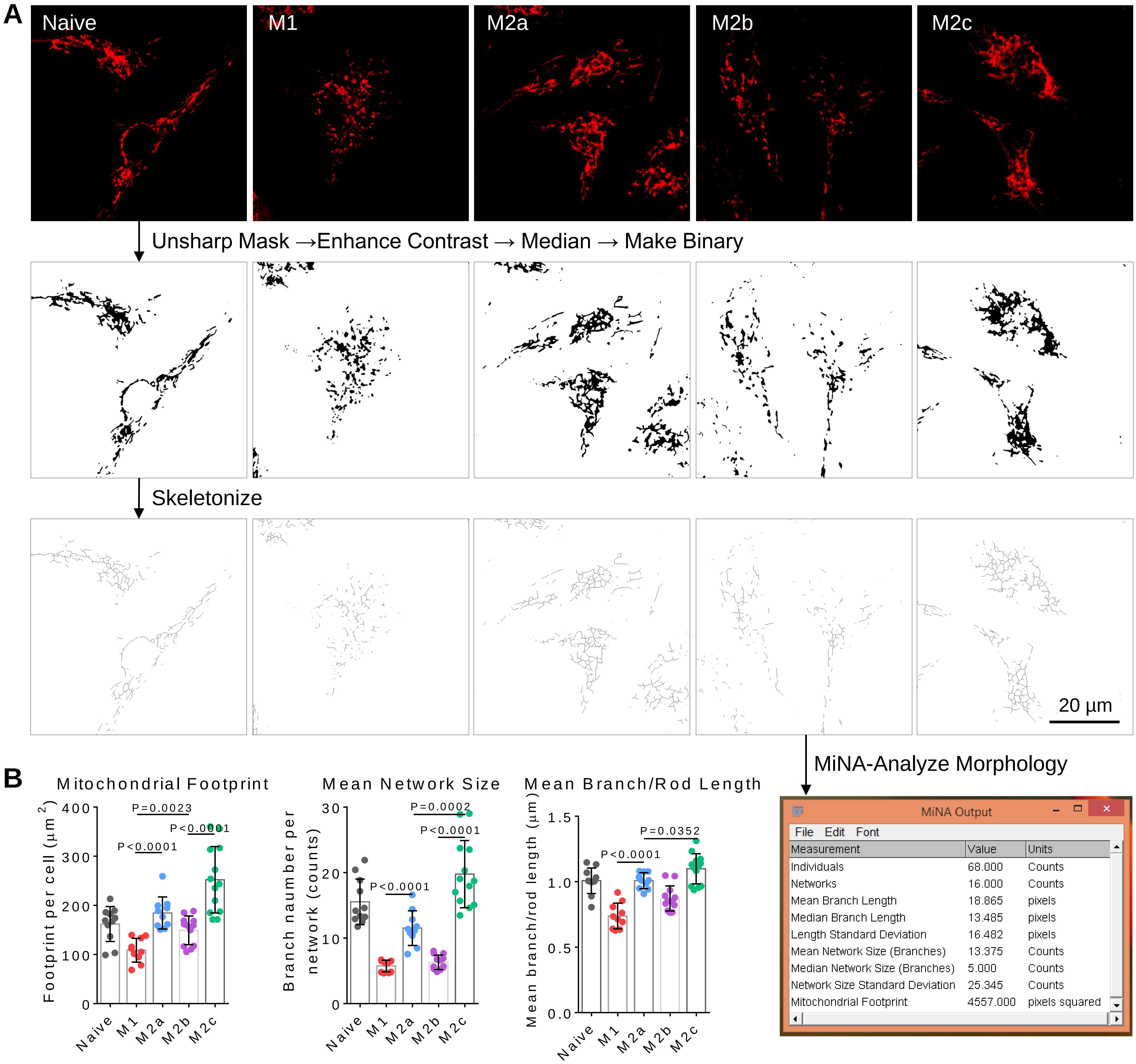

Figure S1. Quantitative analysis of mitochondrial networks in BMDMs. (A) We used ImageJ macro tool to binarize and skeletonize the mitochondrial networks of BMDMs (naive, M1, M2a, M2b, and M2c) from C57BL/6 mice. Then we used the MiNA function to calculate the values of footprints, network size, and branch length. Scale bar, 20 µm. **(B)** Quantitative analysis of the mitochondrial organization in BMDMs (footprints, network size and branch length). Dots are data from individual cells (n≥10).

Figure S2

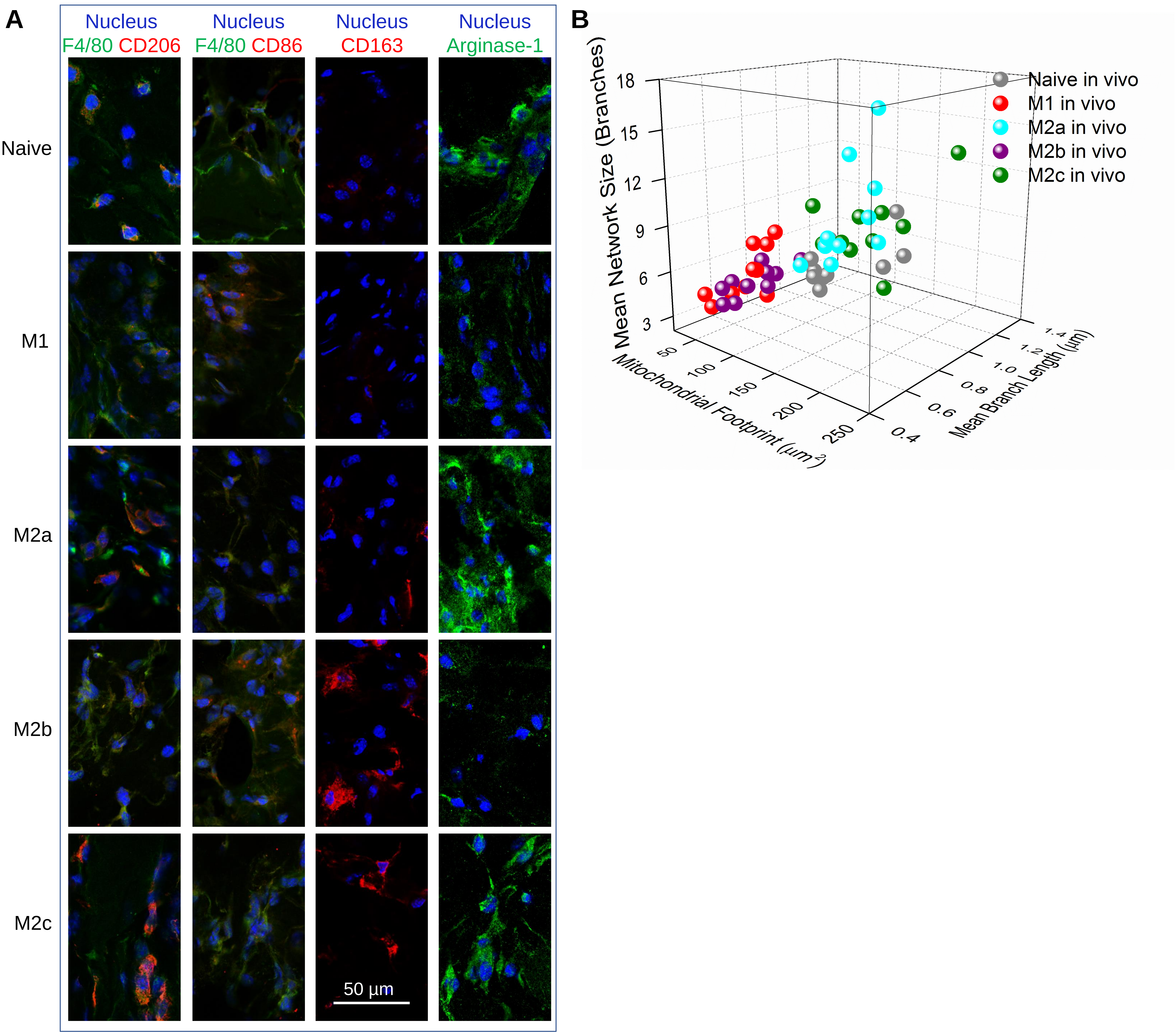

Figure S2. Identification of macrophage activation status in Matrigel plugs. (A) Immunofluorescence labeled imaging of surface markers CD206 (red color in the first column), CD86 (red color in the second column), CD163 (red color in the third column), and intracellular arginase-1 expression (green color in the fourth column) on the fixed cells in Matrigel plugs. Macrophages are stained by F4/80 (green color in the first two columns) marker and nuclei are stained with Hoechst 33342 (blue color). Scale bar, 50 µm. **(B)** 3D scatter plots from mitochondrial network analysis (footprints, network size, and branch length) performed on Matrigel plug-recruited macrophages from C57BL/6 mice (naive *in vivo*, M1 *in vivo*, M2a *in vivo*, M2b *in vivo*, and M2c *in vivo*). The coordinates of each dot represent the feature values of individual cells (n≥10).

Figure S3

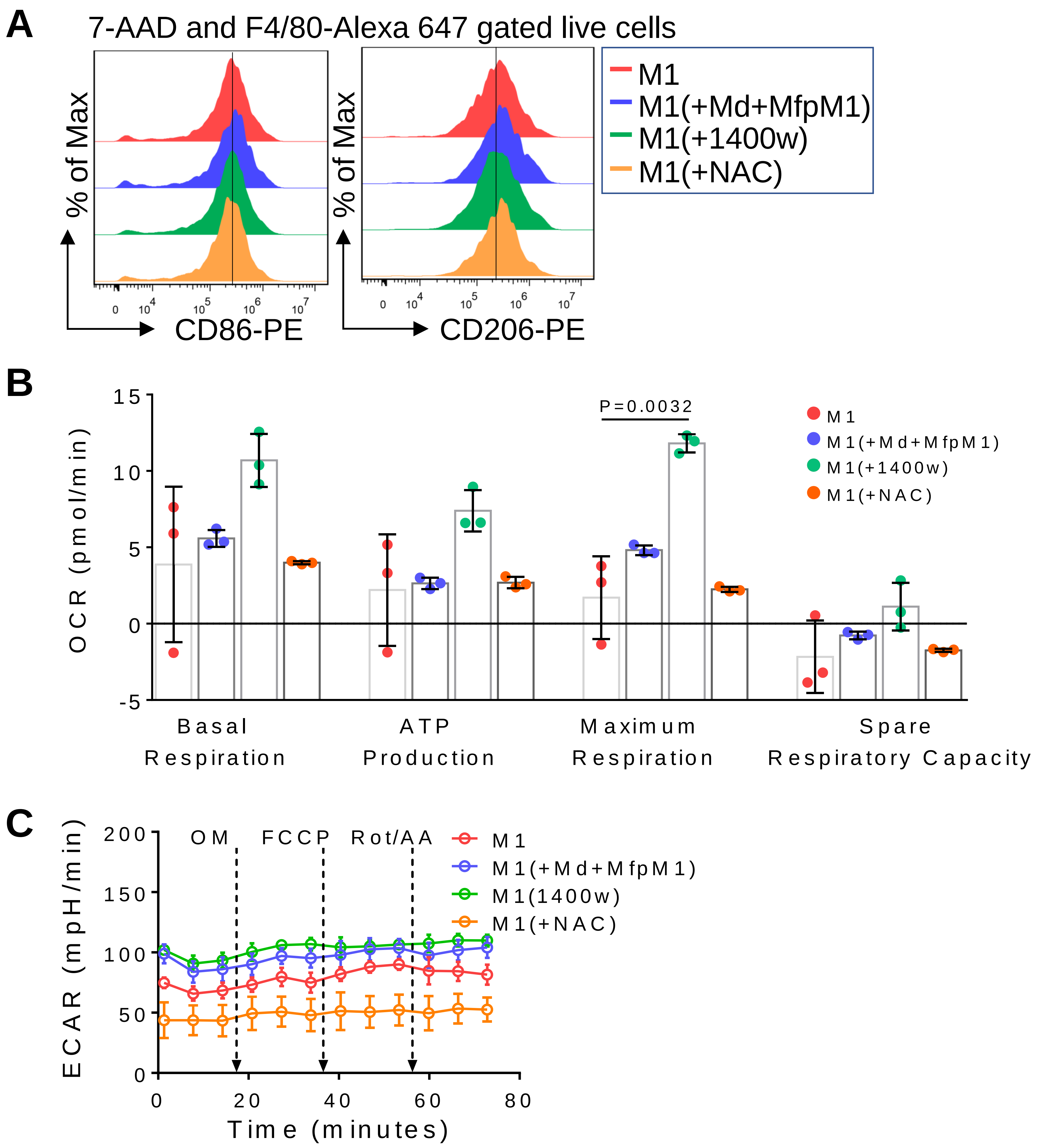

Figure S3. Surface marker and metabolic profile of M1 macrophages under treatments. (A) Flow cytometer was used to evaluate CD86-PE, CD206-PE expression of macrophages (M1, M1[+Mdivi-1+MfpM1], M1[+1400w], and M1[+NAC]). **(B)** Changes of OXPHOS capability during M1 polarization with interventions (control, [+Md+MfpM1], [+1400w], and [+NAC]). **(C)** Changes of ECAR during M1 polarization with interventions (control, [+Md+MfpM1], [+1400w], and [+NAC]).

Figure S4

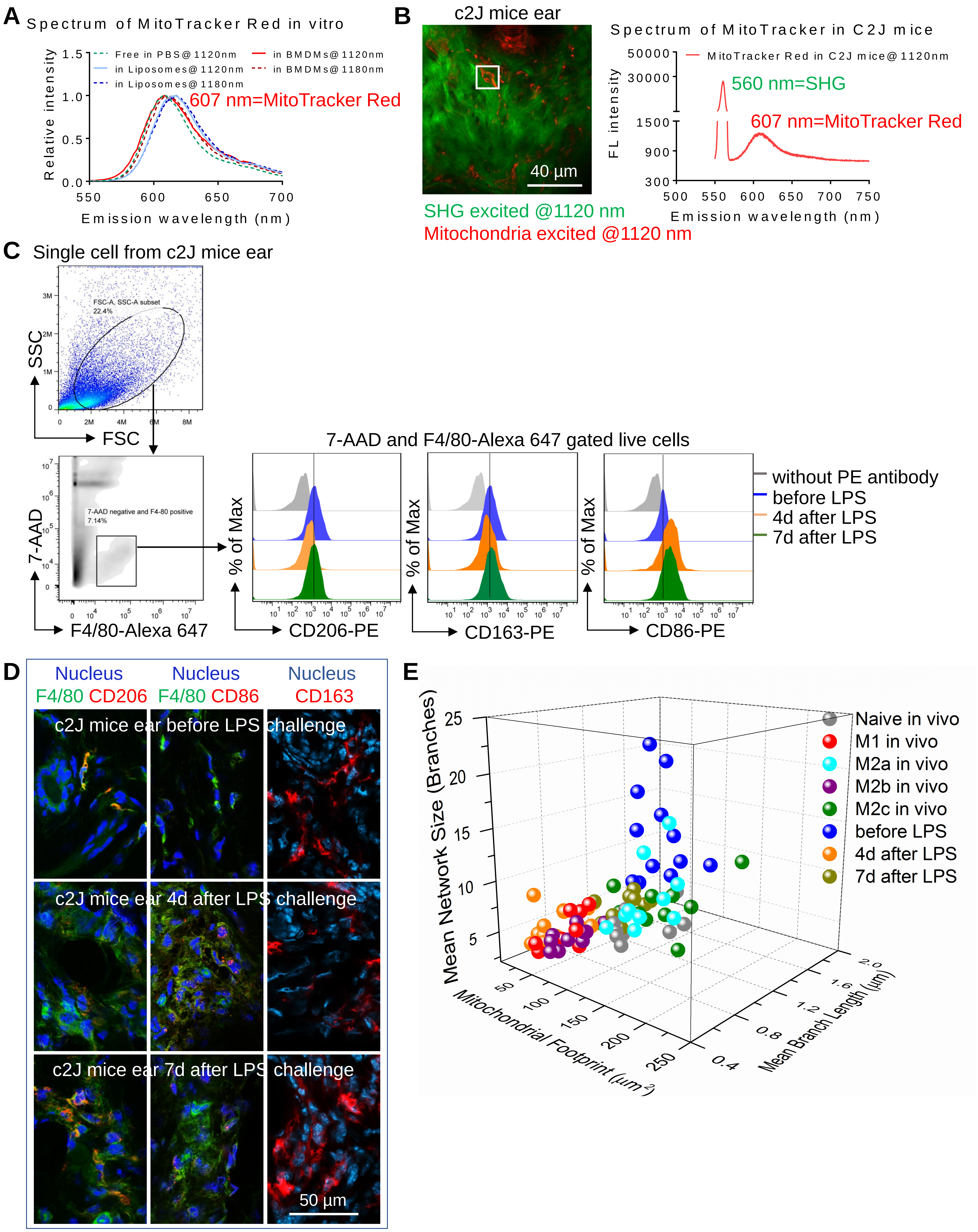
A
Spectrum of MitoTracker Red in vitro
Relative intensity
Free in PBS@1120nm
in Liposomes@1120nm
in Liposomes@1180nm
in BMDMs@1120nm
in BMDMs@1180nm
607 nm=MitoTracker Red
Emission wavelength (nm)
B
c2J mice ear
40 µm
SHG excited @1120 nm
Mitochondria excited @1120 nm
Spectrum of MitoTracker in C2J mice
MitoTracker Red in C2J mice@1120nm
560 nm=SHG
607 nm=MitoTracker Red
FL intensity
Emission wavelength (nm)
C
Single cell from c2J mice ear
FSC-A, SSC-A subset
22.4%
SSC
FSC
7-AAD negative and F4-80 positive
7.14%
7-AAD
F4/80-Alexa 647
7-AAD and F4/80-Alexa 647 gated live cells
% of Max
CD206-PE
CD163-PE
CD86-PE
without PE antibody
before LPS
4d after LPS
7d after LPS
D
Nucleus
F4/80 CD206
Nucleus
F4/80 CD86
Nucleus
CD163
c2J mice ear before LPS challenge
c2J mice ear 4d after LPS challenge
c2J mice ear 7d after LPS challenge
50 µm
E
Mean Network Size (Branches)
Mitochondrial Footprint (µm²)
Mean Branch Length (µm)
Naive in vivo
M1 in vivo
M2a in vivo
M2b in vivo
M2c in vivo
before LPS
4d after LPS
7d after LPS

Figure S4

Figure S4. Identification of macrophage activation status in c2J mice ears before/after LPS challenge. (A) Two-photon fluorescence spectra of MitoTracker dyes in PBS solution (green-dashed curve), in the mitochondria of BMDMs (red-solid and deep red-dashed curves), and in the liposomes (blue-solid and blue-dashed curves). The excitation wavelength is either 1120 nm or 1180 nm. **(B)** Two-photon excited red fluorescence of MitoTracker dyes (red color) and second harmonic generation (SHG, green color) signals in the ear of c2J mice. The excitation wavelength was at 1120 nm. Scale bar, 40 μm. The two-photon excited emission spectra of the region in white square showed a 607 nm peak of red fluorescence. **(C)** To identify the phenotype of macrophages in c2J mice ear, mice ear was harvested and digested to get the single-cell suspension before LPS challenge, and on the 4th or 7th-day after LPS challenge. Then, a flow cytometer was used to assess CD86, CD206, and CD163 expression of F4/80-Alexa 647 positive macrophages. **(D)** Immunofluorescence labeled imaging of surface markers CD206 (red color in the first column), CD86 (red color in the second column), and CD163 (red color in the third column) on fixed cells in c2J mice ear harvested before LPS challenge and on the 4th or 7th-day after LPS challenge. Macrophages are stained by F4/80 (green color) marker and nuclei were stained with Hoechst 33342 (blue). Scale bar, 50 μm. **(E)** 3D scatter plots of mitochondrial organization parameters in macrophages from Matrigel plugs (Naive *in vivo*, M1 *in vivo*, M2a *in vivo*, M2b *in vivo*, and M2c *in vivo*) and c2J mice ear (before LPS, on the 4d after LPS, and 7d after LPS). Dots are data of individual cells ($n \geq 10$).

Figure S5

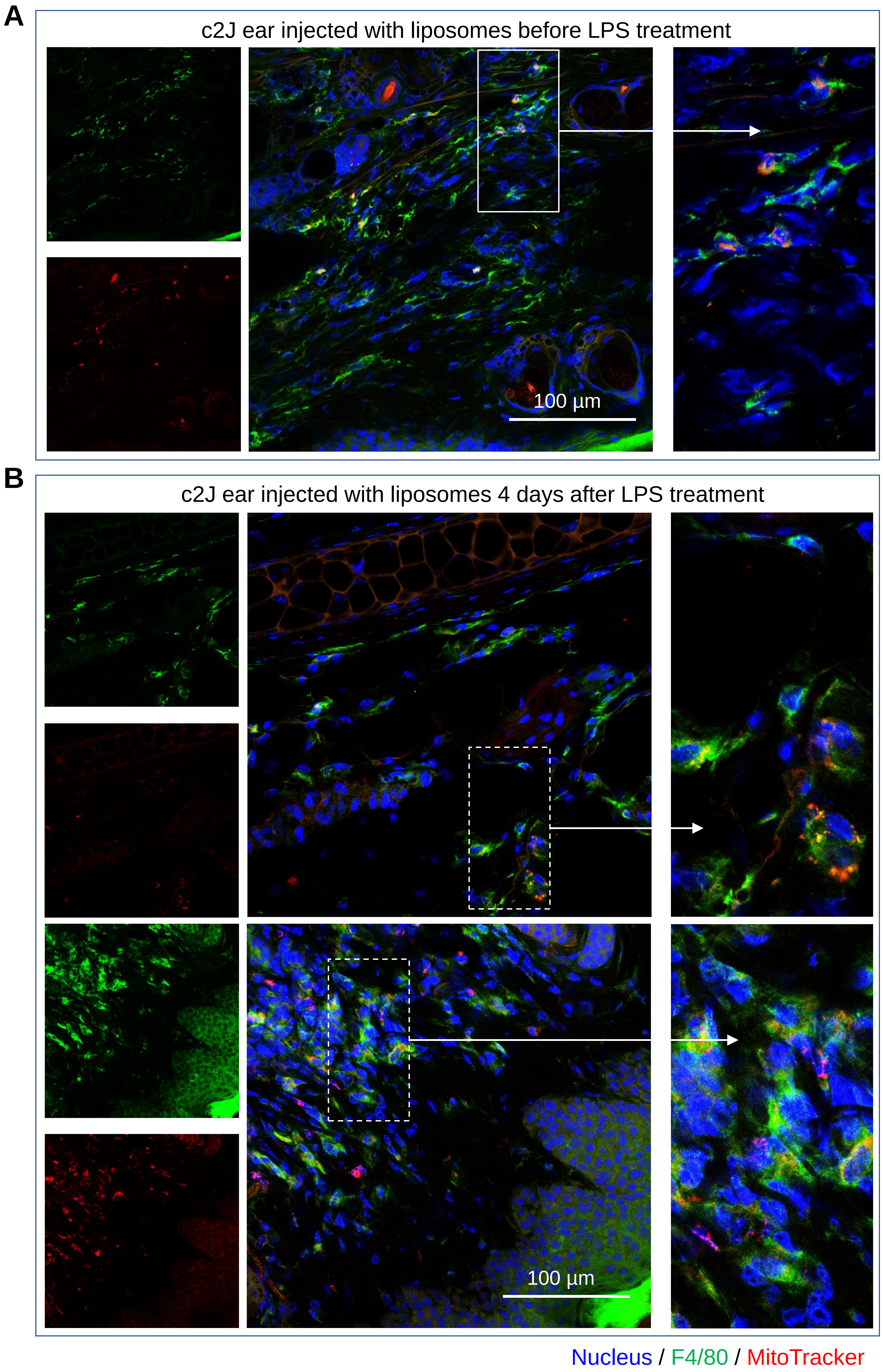

Figure S5. Labeling specificity of MitoTracker-loaded liposomes in c2J mice. Immunofluorescence was used to identify the liposome labeling specificity in c2J mice ear harvested before LPS challenge **(A)**, and on the 4th-day after LPS challenge **(B)**. Cells labeled by MitoTracker-loaded liposomes (red color) were macrophages recognized by F4/80 staining (green color), and their nuclei were stained with Hoechst 33342 (blue). Scale bar, 100 µm.

Figure S6

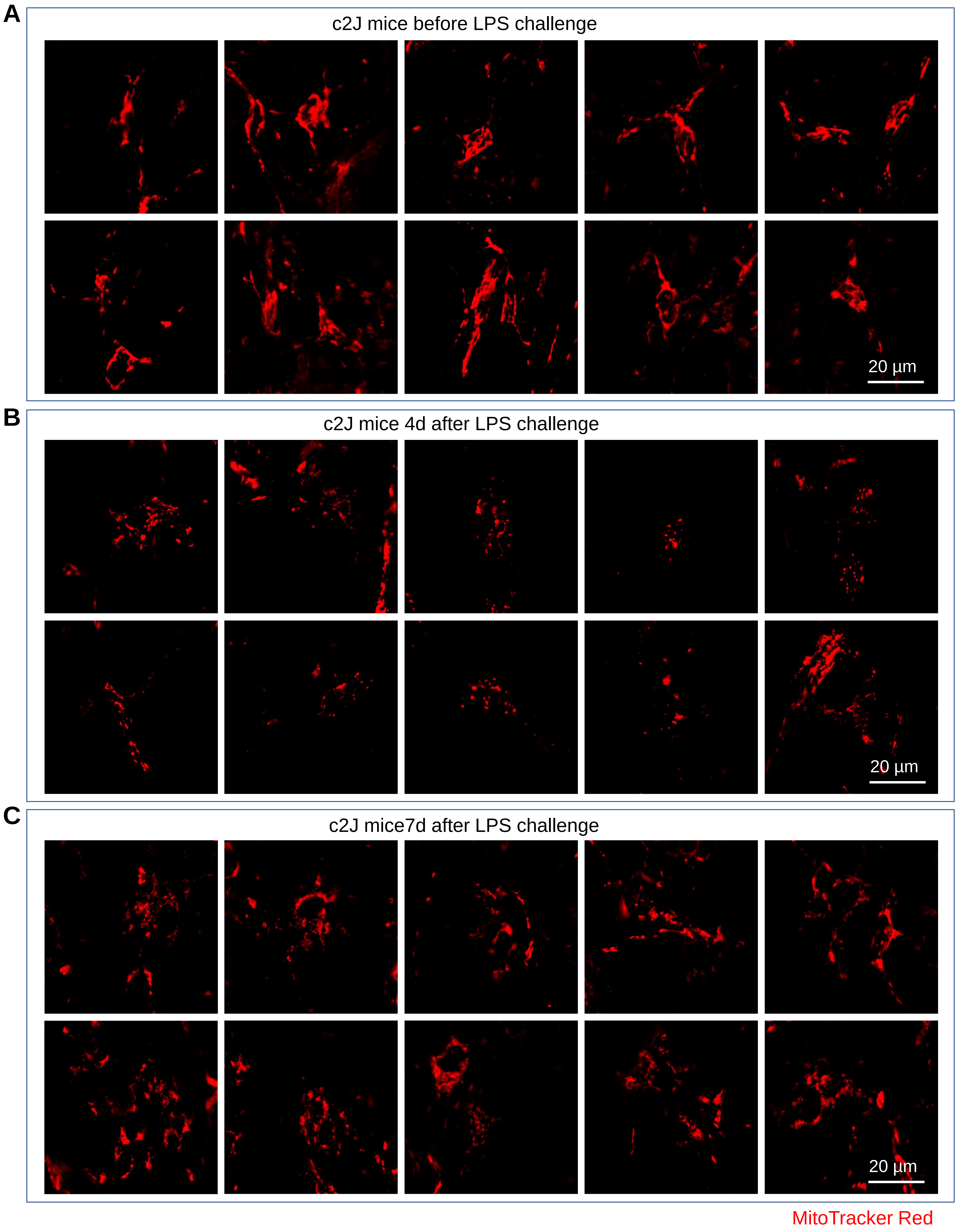

Figure S6. Representative images of mitochondria in the ear of c2J mice before/after the LPS challenge. Two-photon fluorescence images of mitochondria (red) in c2J mice ear before LPS challenge **(A)**, and on the 4th-day **(B)** or 7th-day **(C)** after LPS challenge. The excitation wavelength was 1120 nm. Scale bar, 20 µm.

Figure S7

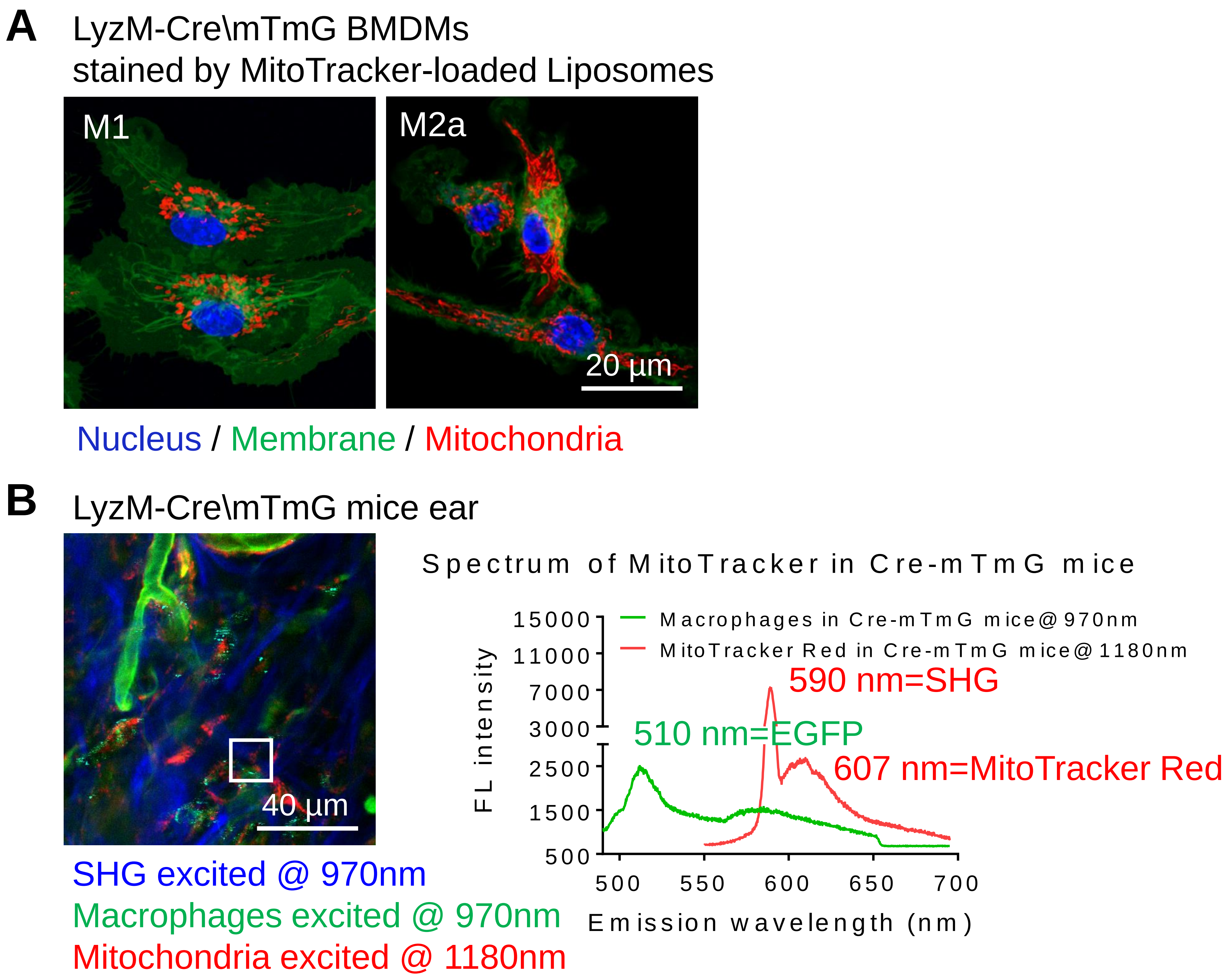

Figure S7. Characterization of MitoTracker-loaded liposomes in LyzM-Cre\mTmG mice. (A) Fluorescence confocal images of the mitochondria (red color, MitoTracker Red CMXRos), nuclei (blue color, Hoechst 33342), and cellular membranes (green color, EGFP) of BMDMs (M1, M2a) from LyzM-Cre\mTmG mice under MitoTracker-loaded liposome staining. Scale bar, 20 µm. **(B)** Identification of red fluorescent cells in LyzM-Cre\mTmG mice using two-photon excited microscopy and spectra. The red color represents the 1180 nm excited two-photon fluorescence of MitoTracker Red. The green color represents the 970 nm excited green fluorescence of EGFP. Blue color represents the 970 nm excited SHG of collagens. Scale bar, 40 µm.
